# What the near Future Holds for Sacubitril/Valsartan: A Summary of Major Ongoing Studies

**DOI:** 10.3390/jcdd9020054

**Published:** 2022-02-10

**Authors:** Hisham A. Badreldin, Nasser Aldosari, Lama Alnashwan, Taif Almutairi, Nada Yousif, Khalid Alsulaiman, Ohoud Aljuhani, Awatif Hafiz, Omar Alshaya

**Affiliations:** 1 King Abdullah International Medical Research Center, Department of Pharmacy Practice, College of Pharmacy, King Saud Bin Abdulaziz University for Health Sciences, King Abdulaziz Medical City, Riyadh 11481, Saudi Arabia; aldawsarina97@gmail.com (N.A.); lama.ibra123@gmail.com (L.A.); taifrasheed15@gmail.com (T.A.); namyousuf@gmail.com (N.Y.); alsulaimankh@hotmail.com (K.A.); shayao@ksau-hs.edu.sa (O.A.); 2 Department of Pharmacy Practice, Faculty of Pharmacy, King Abdulaziz University, Jeddah 21589, Saudi Arabia; oaljuhani@kau.edu.sa (O.A.); whafidh@kau.edu.sa (A.H.)

**Keywords:** sacubitril, valsartan, cardiovascular disease

## Abstract

Early research on neprilysin inhibition showed that sacubitril/valsartan, a combination of the valsartan and the neprilysin inhibitor sacubitril, was superior to enalapril in patients with heart failure with reduced ejection fraction (HFrEF) in the PARADIGM-HF study in 2014. Therefore, for patients with HFrEF, worldwide recommendations have been reformed to include sacubitril/valsartan. In addition, sacubitril/valsartan has been investigated in other cardiovascular disease states, such as patients with heart failure and preserved ejection fraction (HFpEF) and following myocardial infarction (MI) events. In February 2021, the FDA expanded the indication use of sacubitril/valsartan to include the HFpEF patient population based on the results of the PARAGON-HF trial. However, randomized clinical trials post-MI did not show promising results. Sacubitril/valsartan is currently being investigated in many other cardiovascular and non-cardiovascular conditions. This review aims to shed light and summarize the ongoing sacubitril/valsartan registered studies on the United States National Library of Medicine clinical trials registry.

## 1. Introduction

Several pharmacotherapeutic agents emerged over the past few years, and they were able to reshape the modality with which we manage patients with heart failure (HF). Among these agents is the sacubitril/valsartan combination. Sacubitril/valsartan oral combination is an angiotensin receptor neprilysin inhibitor (ARNi) [1]. In 2015, it was approved by the US Food and Drug Administration (FDA) to lower the risk of cardiovascular (CV) death and hospitalization in patients with chronic heart failure with reduced ejection fraction (HFrEF) as a result of the positive outcomes of the PARADIGM-HF trial [2]. The PARADIGM-HF trial compared sacubitril/valsartan to enalapril in patients with HFrEF. The study focused on evaluating the rates of death from CV causes. The PARADIGM-HF study showed that sacubitril/valsartan was significantly more effective in reducing the risk of death from CV causes compared to enalapril [2]. This study was followed by several promising trials, such as the PIONEER-HF trial, which was a multicenter, randomized, double-blinded, and active-controlled trial [3]. This study was conducted to assess the efficacy and safety of adding a neprilysin inhibitor to a renin–angiotensin system inhibitor compared to using a renin–angiotensin system inhibitor alone in patients with HF. This study found that sacubitril/valsartan had better outcomes, such as a higher reduction in the N-terminal pro-brain natriuretic peptide (NT-proBNP) concentration than enalapril therapy [3]. On 1 September 2019, the PARAGON-HF trial concluded that in patients with HF and an ejection fraction of 45% or higher, the addition of sacubitril/valsartan did not result in significantly decreased rates of total hospitalizations for HF or death from CV causes [4]. The role of sacubitril/valsartan has been investigated in patients with acute myocardial infarction (MI) and showed a safety and efficacy profile comparable to ramipril [5]. The demonstrated benefits of sacubitril/valsartan in terms of CV-related death reduction and quality of life improvement make it an appealing treatment option that merits further investigation beyond the HF population. Sacubitril/valsartan has improved insulin sensitivity in obese hypertensive patients [6]. Such findings have stimulated the research on its effect on glycemic control in HF patients with diabetes [7]. After the publication of the PARAGON-HF trial, the question remains if long-term use of sacubitril/valsartan has any prognostic benefits in preventing acute coronary syndrome in HFpEF patients. This question deserves further investigation, especially as a sub-analysis of the PARAGON-HF trial showed beneficial effects in female patients and those with an LVEF between 45% and 57% [4]. Several studies have investigated the role of sacubitril/valsartan in CV remodeling in patients with hypertension, which suggested improvement in LV mass. These findings may not only relate to its blood pressure-lowering characteristics but might also be related to its dual mechanism of action and beyond. This hypothesis and the anti-proliferative effects of sacubitril/valsartan may provide additional benefits in hypertensive patients, which requires further assessment [8]. In terms of its renal protective effect, some data from different populations and animal models suggest that neprilysin inhibition may reduce the risk of end stage disease in chronic kidney disease patients. However, this theory is required to be tested in clinical trials [9]. Due to these suggested benefits, our review aims to shed light on and summarize the ongoing sacubitril/valsartan registered studies in CV and non-CV conditions on ClinicalTrials.gov (accessed on 3 January 2022).

## 2. Materials and Methods

**Figure 1:**
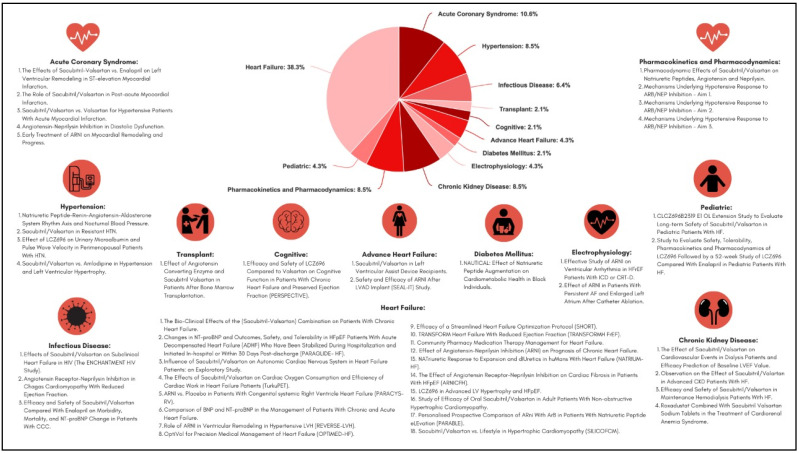
Infographic of the major ongoing sacubitril/valsartan registered studies on ClinicalTrials.gov (accessed on 3 January 2022).

This review included ongoing clinical studies that are registered in the ClinicalTrial.Gov database, operated by the United States National Library of Medicine at the National Institutes of Health, from inception to December 2021 with the keywords ‘sacubitril’ and ‘sacubitril–valsartan.’ We included articles in the English language and are currently actively recruiting or have just completed the recruitment process. We included clinical trials, prospective or retrospective cohorts, meta-analysis, pharmacodynamics studies, and pharmacokinetic studies. The search results from the database yielded 49 articles. Two of these articles were excluded because they did not meet the inclusion criteria. Ultimately, 47 articles were available for review discussing the potential role of sacubitril/valsartan in treating several disease states (Figure 1).

We extracted the following information for each of the included studies: identification number, title, status, conditions, study design, population, interventions, outcome measures, dates, funding, and location, as shown in Supplementary Table S1.

## 3. Results

### 3.1. Acute Coronary Syndrome (ACS)

The term *‘*acute coronary syndrome*’* (ACS) encompasses a variety of clinical presentations, from ST-segment elevation myocardial infarction (STEMI) to non-ST-segment elevation myocardial infarction (NSTEMI) or unstable angina [10]. In the previously published PARADISE-MI, sacubitril/valsartan did not significantly reduce HF rate or CV death compared to ramipril in patients with acute MI [5].

Five ongoing trials examine sacubitril/valsartan*’*s clinical effectiveness in this regard. In the first ongoing trial, sacubitril/valsartan is compared to valsartan for the treatment of acute MI. The investigators use a randomized triple interventional phase 4 study with an estimated enrollment of 200 patients, and the results of this study are expected to be completed by February 2023 [11]. Another study compared sacubitril/valsartan to the traditional ARB or ACE inhibitor algorithms in post-acute MI with diminished left ventricular systolic function using a randomized, open-label design with an estimated enrollment of 100 participants. The study completion date was 1 September 2021 [12]. In addition to the previous studies, the PERI-STEMI study aims to assess whether sacubitril/valsartan prevents adverse left ventricle remodeling more effectively than enalapril in patients with STEMI using a prospective randomized design with an estimated sample size of 376 participants. The expected completion date for this study is June 2026 [13]. The ARNiAMI study, which will be completed by May 2023, seeks to assess whether sacubitril/valsartan affects central hemodynamic parameters during exercise in patients with diastolic dysfunction following acute MI. This study will use a randomized and parallel controlled trial design on 100 participants [14]. Lastly, a total of 280 participants were randomly assigned to receive either sacubitril/valsartan or perindopril to evaluate the efficacy and safety of early sacubitril/valsartan therapy on myocardial remodeling and progression and aerobic exercise capacity in patients who have had an MI. This ongoing study was completed on 30 December 2021 [15].

### 3.2. Heart Failure (HF)

Most of the ongoing studies regarding sacubitril/valsartan use are in patients with HF as shown in Supplementary Table S1. The first study is a multicentered, randomized, and double-blinded clinical trial study that enrolled 800 participants. This study will evaluate the effects of sacubitril/valsartan versus valsartan monotherapy on NT-proBNP levels, clinical outcomes, safety, and tolerability in HFpEF patients after being hospitalized for acute decompensated HF [16]. The second study will enroll 50 participants. It will examine the influence of sacubitril/valsartan on the autonomic cardiac nervous system by determining the heart rate variability in HF patients [17]. The third study is a randomized study that will compare the effects of six weeks of sacubitril/valsartan versus valsartan on cardiac oxygen consumption and cardiac work efficiency in 60 patients with NYHA class II and III HFrEF [18]. The fourth study, the OPTIMED-HF, is a randomized interventional study aiming to measure the rate of change in medical management with beta-blockers and diuretics in patients with ischemic and non-ischemic cardiomyopathy receiving sacubitril/valsartan versus the usual care. This study has an estimated enrollment of 30 participants [19]. The fifth study will be a prospective, randomized, double-blinded, and placebo-controlled crossover study with around 48 patients, in which sacubitril/valsartan will be compared to placebo in terms of the change in exercise capacity and neurohormonal activation in adults with moderate to severe right ventricle dysfunction and NYHA class II or III symptoms [20]. The sixth study investigates the changes in levels of B-type natriuretic peptide (BNP) and the NT-proBNP in outpatients managed in HF clinics under initiated sacubitril/valsartan and directly compares their prognostic values with patients admitted for decompensated HF. The researchers assigned 300 participants to a single group where NT-proBNP and BNP tests will be monitored [21]. The seventh study will be a phase 3, randomized controlled trial in which the researchers will enroll 100 patients to examine the impact of sacubitril/valsartan on reversing cardiac hypertrophy and fibrosis [22].

The eighth study is an interventional open-label non-randomized study with 60 participants, aiming to evaluate the bio-clinical effects of sacubitril/valsartan in treating congestive HF patients. The investigators also studied the side effects of the drug by assessing the renal function and serum electrolytes [23]. The ninth study, the SHORT study, compares two arms in patients with HFrEF. First, the standard arm is ACEi/ARB and low-dose beta-blocker in the first visit. Both are titrated in the second and third visits, while changing ACEi/ARB to sacubitril/valsartan 100 mg by the fourth visit and continuing to titrate each visit. The second experimental arm consists of a low-dose beta-blocker and sodium-glucose cotransporters-2 (SGLT-2) inhibitor and sacubitril/valsartan from the first visit, which is considered an accelerated protocol [24]. The tenth study, the TRANSFORMHFrEF study, targets patients with HFrEF and intends to assess the current guideline-directed medical therapy in this patient population. This study population is 3072 participants [25]. The eleventh study is an open-label clinical trial with 83 participants. Again, those participants are split into two groups. The first group will be managed by community pharmacists with HF medication therapy management training, and the other group will be treated using the standard modality [26]. According to a different study, the effect of angiotensin-neprilysin inhibition on the prognosis of chronic HF will be studied in 340 patients. They will be enrolled in this randomized controlled multicenter clinical trial and randomly assigned to the ARNi group or ACEi/ARB group to evaluate the risk of death due to all causes, cardiac deaths, and re-hospitalization due to HF one, three, and six months after recruitment [27]. In the same way, The NATRIUM-HF trial, which is a multicenter interventional study with an estimated enrollment of 230 participants, is designed to measure the renal response to intravascular fluid expansion and diuretic after the use of sacubitril/valsartan in patients with HF [28]. Meanwhile, the ARNICFH, a randomized, open-label trial that aims to assess the effect of ARNi on cardiac fibrosis in patients with HFpEF, is currently in phase 2, with an estimated population of 60 participants [29].

Another similar study is designed to assess and compare sacubitril/valsartan and valsartan treatments in patients with advanced LV hypertrophy and HFpEF. It is a phase 2 randomized interventional open-label study with an estimated population of 60 participants [30]. Additionally, another randomized interventional open-label study to explore the effectiveness of oral sacubitril/valsartan in adult patients with non-obstructive hypertrophic cardiomyopathy is currently underway with an estimated enrollment of 44 participants [31]. On the other hand, the SILICOFCM study was designed to compare the benefits of sacubitril/valsartan and lifestyle modifications in patients with hypertrophic cardiomyopathy [32]. Lastly, the PARABLE trial examined the hypothesis that sacubitril/valsartan could improve left atrial structure and function and left ventricular structure and function in asymptomatic HFpEF (stage A/B) patients. It was an interventional, randomized, double-blinded controlled trial that recruited 250 participants [33]. Those studies’ completion dates range from June 2020 to May 2024.

### 3.3. Advanced Heart Failure

A recently published RCT has evaluated the efficacy, safety, and tolerability of sacubitril/valsartan in patients with advanced chronic HF. This trial has included 335 patients with advanced HF, in which they found no differences in NT-proBNP levels in the sacubitril/valsartan treatment arm compared with the valsartan treatment arm [34]. Since this trial did not show that sacubitril/valsartan improves the clinical composite of the number of days alive, out of the hospital, and free from HF events, further trials are needed to examine the use of sacubitril/valsartan in advanced HF patients.

The ENVAD-HF study examines the use of sacubitril/valsartan in HeartMate 3 left ventricular assist device (LVAD) recipients. This study recruited about 60 participants to assess all-cause death, worsening kidney function, hyperkalemia, or symptoms of hypotension [35]. Additionally, the SEAL-IT trial compares the safety and efficacy of sacubitril/valsartan in 50 participants with contemporary, durable, continuous-flow LVAD (CF-LVAD) implantation in comparison with patients taking the standard-of-care oral vasodilator therapy [36]. These studies are considered to be completed by August 2023 and December 2022.

### 3.4. Hypertension (HTN)

Several ongoing studies have been conducted to investigate the use of sacubitril/valsartan on patients with hypertension with several different characteristics. Four ongoing studies compare sacubitril/valsartan in obese, left ventricular hypertrophy, or resistant hypertension patients. Another study is evaluating the effect of sacubitril/valsartan in perimenopausal hypertension women. The first study compares morning doses versus evening doses of sacubitril/valsartan or valsartan from baseline and after seven days of intervention. It is a phase 2/3, randomized interventional study with an estimated enrollment of 160 patients. Additionally, the primary outcome is evaluating mean nocturnal systolic blood pressure [37].

The second study (HEVA) compared sacubitril/valsartan with optimized antihypertensive agents [38]. Furthermore, 264 perimenopausal patients with essential hypertension will be enrolled in the third study to examine the efficacy of the treatment on urinary microalbumin content and pulse wave velocity. As mentioned, the primary goal of this study is to assess blood pressure, urinary microalbuminuria, and pulse wave velocity [39]. Finally, the PARASTRAIN study was created to measure the superior efficacy of ARNi on improving hypertension and left ventricular hypertrophy compared to amlodipine. The estimated population of the PARASTRAIN trial is 120 patients [40]. These studies will be completed from July 2021 to January 2027.

### 3.5. Arrhythmias

Two arrhythmias-related studies examine the heart’s electrical system or activity with sacubitril/valsartan. The first study, APART-AF, has an estimated population of 90 patients. It is a randomized interventional open-label study that aims to compare the effects of sacubitril/valsartan and angiotensin receptor blockers on patients with enlarged left atrium diameters and persistent atrial fibrillation within the aspect of cardiac remodeling. The investigators used echocardiography to assess the size of the left atrium and changes in the atrial structure. They hypothesized that sacubitril/valsartan would reverse the cardiac remodeling after catheter ablation compared with ARB agents [41].

As for the second study, it is a prospective, multicentered, interventional, and open-label study. Its purpose is to evaluate the effects of sacubitril/valsartan on ventricular arrhythmia events in approximately 275 HFrEF patients with an implantable cardioverter-defibrillator or cardiac resynchronization therapy-defibrillator. This study’s primary objective is to assess the occurrence of ventricular arrhythmia and anti-tachycardia pacing events that occurred over six months after starting angiotensin-converting enzyme inhibitor/angiotensin receptor blocker treatments compared with the sacubitril/valsartan treatment [42]. Both studies are estimated to be completed between December 2021 and August 2022.

### 3.6. Diabetes Mellitus

The effect of natriuretic peptide augmentation on cardiometabolic health in black individuals is a phase 2, randomized interventional study with 200 enrolled populations. It hypothesizes an improvement in insulin sensitivity and the resting and exercise energy expenditure with sacubitril/valsartan on African American individuals more than with valsartan alone. The study’s primary outcome examines the change in insulin sensitivity after NP augmentation therapy compared to NP-neutral therapy among black participants by measuring the intravenous glucose tolerance test after 12 weeks of intervention. Furthermore, it is estimated that this study will be completed by 30 August 2026 [43].

### 3.7. Chronic Kidney Disease and End Stage Renal Disease

Four studies have been constructed to investigate the efficacy and safety of sacubitril/valsartan in patients undergoing hemodialysis, peritoneal dialysis, advanced CKD, or suffering from cardiorenal anemia syndrome. The purpose of the ESARHD-HF study is to investigate the safety and efficacy of sacubitril/valsartan in patients diagnosed with HF and undergoing maintenance hemodialysis compared to the use of valsartan alone. This study enrolled 118 patients with a primary outcome of evaluating the left ventricle ejection fraction [44].

Similarly, the second study, with an estimated enrollment of 120 participants, aims to assess the efficacy and safety of sacubitril/valsartan on CV events in patients with HF who are undergoing maintenance hemodialysis and peritoneal dialysis [45]. Additionally, the third study observes the efficacy of sacubitril/valsartan in CKD patients with HF for almost 30 adult patients [46]. The final study will compare the new treatment group of roxadustat combined with sacubitril/valsartan versus the traditional treatment group treated with human recombinant erythropoietin (EPO) plus ACEI or ARB in patients with cardiorenal anemia syndrome [47]. These studies are expected to be completed from June 2019 to June 2023.

### 3.8. Human Immunodeficiency Virus (HIV) and Chagas Cardiomyopathy

Several ongoing trials investigate the use of sacubitril/valsartan in patients with infectious disease events. The first study will assess the use of sacubitril/valsartan to decrease HIV-related HF with a preserved ejection fraction. It is an interventional randomized clinical trial with 50 participants and is currently in phase 2. It aims to study the use of sacubitril/valsartan for heart diseases in HIV patients with HF by examining inflammation blood markers, cardiac tissue type imaging, and magnetic resonance imaging [48]. The second study, PARACHUTE-HF, is an interventional randomized phase 4 study with 900 participants. This study will analyze the efficacy and safety of sacubitril/valsartan compared to enalapril in patients with chronic Chagas cardiomyopathy. As for the primary outcome, it will be analyzed by comparing the delayed time to the occurrence of CV death, delayed time to the occurrence of the first HF hospitalization event, or a relative change in NT-proBNP from baseline to week 12 [49]. The third study, ANSWER-HF, is a phase 3, prospective, double-blinded, and randomized clinical trial at a single center with 200 participants. This study evaluates the benefit of sacubitril/valsartan compared with enalapril in patients with HFrEF caused by Chagas cardiomyopathy. The study’s primary endpoint will measure the changes in left ventricular ejection fraction using transthoracic echocardiography [50]. These three studies are estimated to be completed by December 2022–2024.

### 3.9. Transplant

There is a randomized, open-label interventional study with a population of 90 participants. It will examine the effect of ACE inhibitors versus sacubitril/valsartan in patients with bone marrow transplants by measuring the CV and endothelial parameters to search for a more potent agent to prevent cardiotoxicity immediately after bone marrow transplantation. Furthermore, the endpoint measures the effect of treatment on left ventricular function by calculating the ejection fraction using 3D echocardiography and examining the global longitudinal strain through speckle tracking echocardiography. Moreover, the investigators will measure arterial stiffness by evaluating pulse wave velocity. The study was estimated to be completed by 1 September 2021 [51].

### 3.10. Cognitive Function

A study aims to check cognitive functions in chronic HF patients with preserved ejection fraction using sacubitril/valsartan therapy versus valsartan alone. It is a phase 3, multicenter, randomized, double-blinded, and active-controlled study. The study’s primary outcome measures patients’ cognitive state by presenting a global cognitive composite z score change. This score includes cognitive domains, such as memory, attention, and executive functions. Moreover, 592 participants enrolled in this study, and all essential documents are expected to be completed by 28 March 2022 [52].

### 3.11. Pediatrics

Two studies assess the pediatric patient safety, tolerability, pharmacokinetics, and pharmacodynamics of sacubitril/valsartan. The purpose of the PANORAMA-HF trial is to investigate the long-term safety and tolerability of LCZ696 in pediatric patients [53]. For the second trial, part 1 assesses the body’s absorption, distribution, and elimination of LCZ696, which will help determine the appropriate dosage for part 2, in which the study aims to compare the effectiveness and safety of LCZ696 with enalapril. Furthermore, this study enrolled 393 patients to analyze several pharmacokinetic and pharmacodynamic factors that are related to LCZ696 [54]. These studies are considered to be completed by December 2021–2022.

### 3.12. Chemotherapy Induced Left Ventricular Dysfunction

Two studies are currently ongoing investigating the role of LCZ969 as either a cardioprotective during chemotherapy treatment or its feasibility and tolerability in adult cancer survivors. Treating Heart Dysfunction Related to Cancer Therapy With Sacubitril/Valsartan (TREAT-HF) is an ongoing randomized interventional trial estimated to be completed by 30 June 2024. The study aims to enroll 30 adults with stage B HF who were between the ages of 18 and 39 at the time of cancer diagnosis. Patients will be included if they are symptom-free during enrollment and have received anthracycline chemotherapy. The study will evaluate the recruitment feasibility, efficacy, and tolerability of sacubitril/valsartan among adult cancer patients with stage B HF [55]. The second trial, which is anticipated to be completed by 14 September 2025, is the Prevention of Cardiac Dysfunction During Breast Cancer Therapy (PRADAII). This is a randomized multicenter placebo-controlled double-blinded trial and aims to recruit 214 patients and follow them for 18 months with cardiac imaging, blood labs, adverse effects, and quality of life. The study hypothesizes that sacubitril/valsartan used concomitantly during anthracycline-containing chemotherapy for breast cancer treatment will prevent cardiac dysfunction, measured by cardiac magnetic resonance imaging (CMR) [56].

### 3.13. Pharmacokinetics and Pharmacodynamics

Several ongoing trials examine the pharmacokinetics and pharmacodynamics of combined angiotensin receptor blockade (ARB)/neprilysin (NEP) inhibitors. The first study is a randomized, double-blinded, and crossover-design study with the primary objective of testing the hypothesis that the substance P affects ARB/NEP inhibition on blood pressure, natriuresis, and diuresis at initiation after up-titration in 80 subjects with stable HF [57]. The second study is a randomized crossover study with 80 participants with stable HF. It aims to examine whether endogenous bradykinin is involved in the effects of ARB/NEP inhibition on blood pressure, natriuresis, and diuresis at onset and at a steady-state [58]. The third study examines the pharmacodynamic impact of sacubitril/valsartan on natriuretic peptides, angiotensin, and neprilysin. It was performed on 40 patients with HF treated with sacubitril/valsartan in gradually increasing doses over eight weeks [59]. Finally, the last study will enroll 32 participants to investigate whether combined ARB/NEP enhanced the effects of exogenous bradykinin, substance P, and brain natriuretic peptide on blood flow or endothelial tissue-type plasminogen activator release compared with the use of ARB monotherapy [60]. The estimated completion of the mentioned studies will be from October 2023 to December 2024.

## 4. Discussion

To date, the most substantial evidence for the use of sacubitril/valsartan in CVD, particularly HF, comes from the PARADIGM-HF and the PARAGON-HF [2,4]. In these studies, patients treated with valsartan/sacubitril had substantially improved CVD outcomes compared with the current standard of care (ARB or ACEI alone). Hence, as observed above, the major focus of research for the use of LCZ969 remains in the realm of HF.

Additionally, based on previous data and analyses, sacubitril/valsartan showed its tremendous potential effect on reducing CVD risk factors, particularly hypertension. The analysis of PARADIGM-HF showed a statistically significant mean reduction in systolic BP of 3.2 ± 0.4 mmHg from baseline at randomization in the sacubitril/valsartan arm compared with the enalapril arm (*p* < 0.001). However, initial studies investigating neprilysin inhibitors alone did not carry any clinical benefits in both HF and hypertension patients [61]. Therefore, the proposed mechanism behind the benefit on the vascular endothelium arises from the combination of a neprilysin inhibitor with an angiotensin receptor block, leading to disinhibition of potent vasoconstrictor peptides such as angiotensin I and II [62]. Prior to the PARADIGM-HF, the efficacy of *Entresto* on diastolic blood pressure reduction was seen in a dose-dependent manner [63,64]. Therefore, upcoming trials in the pipeline, as discussed above, currently question its effect on nocturnal hypertension and add-on therapy in combination with first-line therapeutic agents such as calcium channel blockers or hypertension subsets such as diabetics and CKD. However, as with all these pros of sacubitril/valsartan in both HF and hypertension, the limitations associated with ARNi should be acknowledged as well. Upcoming trials, particularly in the HTN population, should be further evaluated carefully for the risk of symptomatic hypertension, hyperkalemia, and worsening renal failure in addition to its clinical utility.

As of now, data available provide a positive impact on its effect on cardiac remodeling and protection as well as on vascular and endothelium dysfunction. Numerous clinical trials are evolving across CVD benefits, CVD risk factors, and non-CV disease states, suggesting that LCZ969 may carry additional mechanistic insights that we are not aware of. However, one can conclude that their potential benefits might subside beyond what is seen due to the neurohormonal RAAS and SNS blockade. Expanding indications based on upcoming data results of these ongoing trials and the development of more novel ARNi’s to target different subsets of CV disease remains a promising question to look for in the future.

## 5. Conclusions

Sacubitril/valsartan is currently being investigated in many CV and non-CV conditions. This review aims to shed light and summarize the ongoing sacubitril/valsartan registered studies on ClinicalTrials.gov (accessed on 3 January 2022). The highlighted studies could change the management of several disease states such as advanced HF, hypertension, chronic kidney disease, HIV, and many other disease states.

## Data Availability

Not applicable.

## Supplementary Materials

The following are available online at https://www.mdpi.com/article/10.3390/jcdd9020054/s1, Table S1: Summary the ongoing sacubitril/valsartan registered studies on ClinicalTrials.gov (accessed on 3 January 2022).
