# Supplementary material for: What the near Future Holds for Sacubitril/Valsartan: A Summary of Major Ongoing Studies
Source: J Cardiovasc Dev Dis. 2022 Feb 10;9(2):54. doi: 10.3390/jcdd9020054 (PMC8875386; doi:10.3390/jcdd9020054)
Supplement: Supplementary file 1 [file jcdd-09-00054-s001.zip › jcdd-1563174-supplementary.pdf]

Table S1. Summary the ongoing sacubitril/valsartan registered studies on ClinicalTrials.gov.

#	ID	Title	Status	Conditions	Study Design	Population	Interventions	Outcome measures	Dates	Funding	Location	Remarks
Acute Coronary Syndrome												
1	• NCT number: NCT05060588<br><br>• Other Ids: Sacu-HPT	Sacubitril/ Valsartan Versus Valsartan for Hypertensive Patients With Acute Myocardial Infarction	Not yet recruiting	•Myocardial Infarction<br><br>•Hypertension	Study Type: Interventional<br><br>Phase: Phase 4<br><br>Study Design:<br>•Allocation: Randomized<br><br>•Intervention Model: Parallel Assignment<br><br>•Masking: Triple (Participant, Investigator, Outcomes Assessor)<br><br>•Primary Purpose: Treatment	Enrollment: 200 Participants<br>Age: 18 Years to 75 Years (Adult, Older Adult)<br>Gender: All<br><br>Inclusion Criteria:<br>•Diagnosed with new-onset MI, either STEMI or NSTEMI, according to the fourth universal definition of MI (Thygesen et al. 2019), disease onset within 7 days.<br>•Patients are previously diagnosed with essential hypertension or newly diagnosed with essential hypertension.<br>•Capable of giving signed informed consent, which includes compliance with the requirements and	•Drug: Sacubitril/ Valsartan 49/51mg/ Tab<br><br>•Drug: Valsartan 80mg/Tab	Primary Outcome:<br>•Rate of MACE events no.1 by phone calls and questionnaires<br>•Rate of MACE events No.2 by phone calls and questionnaires<br>•Rate of MACE events No.3 phone calls and questionnaires<br><br>Secondary Outcome:<br>•Left ventricular ejection fraction(LVEF) by echocardiography<br>•Rate of post infarction angina by following up in the clinic<br>•The rate of heart failure occurrence by following up in the clinic<br>•Left ventricular end-diastolic volume(LVEDV) by echocardiography	Study Start: October 2021<br><br>Primary Completion: October 2022<br><br>Study Completion: February 2023<br><br>First Posted: September 29, 2021<br><br>Results First Posted: No Results Posted<br><br>Last Update Posted: September 29, 2021	•Qingdao Central Hospital<br><br>Funder Type:<br>•Other	•Mengmei Li, Qingdao, Shandong, China	This study aims to assess the effect of Sacubitril/Valsartan on short-term prognosis in hypertensive patients with acute myocardial infarction compared against Valsartan.

						restrictions listed in the informed consent form (ICF) and the protocol. (exclusion details present in the study.)						

2	• NCT number: NCT03893435<br><br>• Other IDs: YIG01201902<br><br>• Title Acronym: RSVP-AMI	The Role of Sacubitril/ Valsartan in Post-acute Myocardial Infarction	Recruiting	• Acute Myocardial Infarction	• Study Type: Interventional<br><br>• Phase: Not Applicable<br><br>• Allocation: Randomized<br><br>• Intervention Model: Parallel Assignment<br><br>• Masking: None (Open Label)<br><br>• Primary Purpose: Treatment	• Enrollment: 100<br><br>• Ages: 18 Years and older<br><br>• Gender: All<br><br>• Inclusion:<br>- Post-AMI patients who underwent successful PPCI and LVEF ≤40%..<br><br>(exclusion details present in the study.)	•Intervention: Sacubitril/Valsartan<br><br>•Comparator: Valsartan	• Primary outcome:<br>1. One week major adverse cerebrovascular and cardiovascular events (MACCE) [Time Frame: 1 week after AMI]<br>2. Twenty four weeks major adverse cerebrovascular and cardiovascular events (MACCE) [Time Frame: 24 weeks after AMI]<br>3. Change in the ejection fraction during hospital stay, 3 months and 6 months after AMI. [Time Frame: In hospital, 3 months and 6 months after AMI]<br><br>• Secondary outcome:<br>Not provided<br><br>(more details in the study.)	• Study start: December 1, 2018<br><br>• Primary Completion: September 1, 2021<br><br>• Study Completion: September 1, 2021<br><br>• First Posted: March 28, 2019<br><br>• Results First Posted: No Results Posted<br><br>• Last Update Posted: June 1, 2021	.•Funder Type: Other	• Andalusia Hospitals, Alexandria, Egypt	

3	• NCT number: NCT04912167<br><br>• Other IDs: WestChinaH-CVD-003<br><br>• Title Acronym: PERI-STEMI	The Effects of Sacubitril-Valsartan vs Enalapril on Left Ventricular Remodeling in ST-elevation Myocardial Infarction	Not yet recruiting	• STEMI	• Study Type: Interventional<br><br>• Phase: Phase 3<br><br>• Allocation: Randomized<br><br>• Intervention Model: Parallel Assignment<br><br>• Masking: Single (Outcomes Assessor)<br><br>• Primary Purpose: Treatment	• Enrollment: 376<br><br>• Ages: 18 Years to 75 Years<br><br>• Gender: All<br><br>• Inclusion:<br>- First-time ST-segment elevation myocardial infarction based on the newest ESC guidelines<br><br>- Timely primary percutaneous coronary intervention within 12 hours from onset<br><br>- Written informed consent acquired<br><br>(exclusion details present in the study.)	•Intervention: ARNI-Sacubitril-Valsartan<br><br>•Comparator: ACEI-Enalapril	• Primary outcome:<br>1. LV remodeling index on CMR [Time Frame: 6 months]<br><br>• Secondary outcome:<br>1. left ventricular (LV) ejection fraction [Time Frame: 6 months]<br>2. global peak LV longitudinal strain [Time Frame: 6 months]<br>3. myocardial fibrosis [Time Frame: 6 months]<br>4.Time to the first occurrence of a composite endpoint of adverse clinical events [Time Frame: up to approximately 60 months]<br>(more details in the study.)	• Study start: November 2021<br><br>• Primary Completion: March 2022<br><br>• Study Completion: June 2026<br><br>• First Posted: June 3, 2021<br><br>• Results First Posted: No Results Posted<br><br>• Last Update Posted: September 29, 2021	• West China Hospital.<br><br>• Funder Type: Other	• West China Hospital, Chengdu, Sichuan, China	

4	• NCT number: NCT04149990<br><br>• Other IDs: Version 1.4<br><br>• Title Acronym: ARNiAMI	Angiotensin-Neprilysin Inhibition in Diastolic Dysfunction	Recruiting	• Myocardial Infarction<br><br>• Diastolic Dysfunction	• Study Type: Interventional<br><br>• Phase: Phase 2<br><br>• Allocation: Randomized<br><br>• Intervention Model: Parallel Assignment<br><br>• Masking: Quadruple (Participant, Care Provider, Investigator, Outcomes Assessor)<br><br>• Primary Purpose: Treatment	• Enrollment: 100<br><br>• Ages: 50 Years and older<br><br>• Gender: All<br><br>• Inclusion:<br>- Documented ST segment elevation or non ST-myocardial infarction according to current guidelines<br>- Complete revascularization<br>- Age ≥50 years<br>- LVEF ≥45% on echocardiography performed within 72 hours of the MI.<br>- Diastolic dysfunction defined as: Ratio of early diastolic peak mitral inflow velocity (E) to early mitral annulus diastolic velocity (e') ratio > 8 and at least moderate LA dilatation (LA volume index>34 mL/m2).	•Intervention: Entresto<br><br>•Comparator: Placebo	• Primary outcome:<br>1. Central hemodynamics [Time Frame: 26 weeks]<br><br>• Secondary outcome:<br>1. Cardiac MRI [Time Frame: 26 weeks]<br>2. Percentage of premature ventricular beats. [Time Frame: 6 months]<br>3. Biomarkers [Time Frame: 26 weeks]<br>4. Echocardiographic [Time Frame: 26 weeks]<br><br>(more details in the study.)	• Study start: October 12, 2018<br><br>• Primary Completion: October 2022<br><br>• Study Completion: May 2023<br><br>• First Posted: November 4, 2019<br><br>• Results First Posted: No Results Posted<br><br>• Last Update Posted: October 14, 2021	• Jacob Moller<br><br>• Danish Heart Foundation<br><br>• Odense University Hospital<br><br>• Rigshospitalet, Denmark<br><br>.<br>• Funder Type: Other	• Department of Cardiology, Rigshospitalet, Copenhagen, Denmark<br><br>•Department of Cardiology, Odense Universityhospital, Odense, Denmark	

						-Signed informed consent (exclusion details present in the study.)						

5	• NCT number: NCT04342351<br><br>• Other IDs: CRP	Early Treatment of ARNI on Myocardial Remodeling and Progress	Not yet recruiting	• Myocardial Infarction	• Study Type: Interventional<br><br>• Phase: Phase 4<br><br>• Allocation: Randomized<br><br>• Intervention Model: Parallel Assignment<br><br>• Masking: Double (Participant, Investigator)<br><br>• Primary Purpose: Treatment	• Enrollment: 280<br><br>• Ages: 18 Years to 80 Years<br><br>• Gender: All<br><br>• Inclusion:<br>- Acute myocardial infarction (AMI) within 1 months prior to recruitment;<br>- Aged 18 years or over and under 80 years;<br>- Randomized patients will have been hemodynamically stable, SBP ≥100mmHg, no symptomatic hypotension;<br>- NYHA Class Ⅱ-Ⅳ, HFrEF or HFpEF;<br>- Elevated NT-proBNP or BNP at the time of screening;<br>-Peak VO2/kg<16 ml/kg/min by CPET<br>(exclusion details present in the study.)	•Intervention: Sacubitril/Valsartan<br><br>•Comparator: Perindopril	• Primary outcome:<br>1. Peak oxygen consumption (VO2)/kg [Time Frame: 3 months]<br>2. Peak Oxygen Pulse (O2-Pulse) [Time Frame: 3 months]<br>3. LVEF [Time Frame: 3 months]<br><br>• Secondary outcome:<br>1. Peak VO2/kg change [Time Frame: 6 months]<br>2. Peak Oxygen Pulse change [Time Frame: 6 months]<br>3. Ventilatory efficiency (VE/VCO2 slope) change [Time Frame: 6 months]<br>4. LVEF change [Time Frame: 6 months]<br>5. N-terminal Pro-brain Natriuretic Peptide (NT-proBNP) change [Time Frame: Baseline, 3, 6 months]<br>6. The MOS item short form health survey, SF-36 [Time Frame: baseline and 6 months]<br>(more details in the study.)	• Study start: May 1, 2020<br><br>• Primary Completion: May 1, 2021<br><br>• Study Completion: December 30, 2021<br><br>• First Posted: April 13, 2020<br><br>• Results First Posted: No Results Posted<br><br>• Last Update Posted: April 14, 2020	• RenJi Hospital<br>.<br>• Funder Type: Other	• RenJi Hospital, Shanghai JiaoTong University, School of Medicine, Shanghai, Shanghai, China	
Heart Failure												

1	• NCT number: NCT03988634 • Other Id's: CLCZ696DUS01	Changes in NT-proBNP and Outcomes, Safety, and Tolerability in HFpEF Patients With Acute Decompensated Heart Failure (ADHF) Who Have Been Stabilized During Hospitalization and Initiated In- hospital or Within 30 Days Post-discharge (PARAGLIDE-HF)	Recruiting	Heart Failure With Preserved Ejection Fraction (HFpEF)	• Study Type: Interventional<br><br>• Phase: Phase 3<br><br>• Allocation: Randomized<br><br>• Intervention Model: Parallel Assignment<br><br>• Masking: Double (Participant, Investigator)<br><br>• Primary Purpose: Treatment	• Enrollment: 800<br><br>• Age: 40 Years and older (Adult, Older Adult)<br>• Sex: All<br><br>• Inclusion criteria:<br>1. signed informed consent.<br><br>2. ≥40 years of age, male or female.<br><br>3. Hospitalized for or within 30 days following discharge of an acute decompensated HFpEF admission.<br><br>4. Patients with a diagnosis of acute heart failure had to have symptoms and signs of fluid overload.<br><br>5. HFpEF with most recent LVEF >40% (within past 3 months).<br><br>6. Elevated NT-proBNP or BNP at the time of	•Drug: sacubitril / valsartan<br><br>•Drug: valsartan	• Primary outcome: [Time Frame: Baseline to weeks 4 and 8]<br>1. Proportional change in NT-proBNP from baseline to the average of weeks 4 and 8.<br><br>• Secondary outcome:<br>1. Composite hierarchical outcome [Time Frame: Baseline to Weeks 4 and 8]<br>2. Cumulative number of recurrent composite overtime [Time Frame: Overtime]<br>3. Incidences of a composite endpoint of worsening renal function [Time Frame: Overtime]<br>4. Proportional change in NT-proBNP from baseline to Week 8 [Time Frame: Baseline to week 8]<br>5. Proportional change from baseline in hs-Troponin (high sensitivity) at Weeks 4 and 8 [Time Frame: Baseline to weeks 4 and 8]<br><br>(more details in the study.)	• Study Start: June 27, 2019<br><br>• Primary Completion: October 31, 2022<br><br>•Study Completion: October 31, 2022<br><br>• First Posted: June 17, 2019<br><br>• Results First Posted: No Results Posted<br><br>• Last Update Posted: December 6, 2021	• Novartis Pharmaceuticals<br>• Novartis<br><br>• Funder type: Other	• Novartis Investigative Site, Little Rock, Arkansas, United States<br><br>• Novartis Investigative Site, Fresno, California, United States<br><br>• Novartis Investigative Site, Long Beach, California, United States<br><br>• Novartis Investigative Site, Los Angeles, California, United States<br><br>• Novartis Investigative Site, Orange, California, United States	The study evaluated the effects of sacubitril/valsartan vs. valsartan monotherapy in HFpEF (LVEF > 40%) patients after hospitalization for acute decompensated heart failure.

						screening.<br><br>7. Has not taken an ACEi for 36 hours prior to randomization.<br><br>(exclusion details present in the study.)					•Novartis Investigative Site, San Diego, California, United States<br><br>• Novartis Investigative Site, San Pablo, California, United States<br><br>• Novartis Investigative Site, Santa Ana, California, United States<br><br>• Novartis Investigative Site, Littleton, Colorado, United States<br><br>• Novartis Investigative Site, West Hartford, Connecticut,	

											United States<br><br>• and 95 more	

2	• NCT number: NCT04587947 • Other Id's: Entresto	Influence of Sacubitril/ Valsartan on Autonomic Cardiac Nervous System in Heart Failure Patients: an Exploratory Study	Recruiting	• Heart Failure Patients	• Study Type: Interventional<br><br>• Phase: Not Applicable<br><br>• Allocation: N/A<br><br>• Intervention Model: Single Group Assignment<br><br>• Masking: None (Open Label)<br><br>• Primary Purpose: Treatment	• Enrollment: 50<br><br>• Age: 20 Years to 99 Years (Adult, Older Adult)<br><br>• Sex: All<br><br>• Inclusion criteria:<br>1. written informed consent.<br><br>2. Patients with symptomatic severe heart failure with reduced ejection fraction (LVEF ≤40%,)<br><br>3. Age > 20 years<br><br>4. Sinus rhythm<br><br>5. Suitable for a drug conversion from AT1/ACE inhibitors to sacubitril/valsartan<br><br>(exclusion details present in the study.)	• Drug: sacubitril / valsartan	• Primary outcome: [Time Frame: 24 months]<br>1. Changes in heart rate variability.<br>2. Changes in left ventricular ejection fraction (%).<br>3. Changes in mitral insufficiency grade.<br>4. Changes in serum NT-proB-Type natriuretic peptide (ng/l).<br>5. Changes in Serum creatinine level (mg/dl).<br>6. Changes in glomerular filtration rate (ml/min).<br>7. Changes in serum potassium level (mmol/l).<br><br>(more details in the study.)	• Study Start: April 1, 2020<br><br>• Primary Completion: April 2022<br><br>• Study Completion: April 2022<br><br>• First Posted: October 14, 2020<br><br>• Results First Posted: No Results Posted<br><br>• Last Update Posted: October 14, 2020	• St. Josefs-Hospital Wiesbaden GmbH<br><br>• Funder type: Other	• St. Josefs-Hospital Wiesbaden GmbH, Wiesbaden, Germany	The study examines the effects of Sacubitril/Valsartan on the autonomic cardiac nervous system in heart failure patients.

3	•NCT number: NCT03300427<br><br>•Title Acronym: TurkuPET	The Effects of Sacubitril/ Valsartan on Cardiac Oxygen Consumption and Efficiency of Cardiac Work in Heart Failure Patients (TurkuPET)	Recruiting	• Heart Failure	Study Type: Interventional<br><br>• Phase: Phase 4<br><br>• Allocation: Randomized<br><br>• Intervention Model: Parallel Assignment<br><br>• Masking: Quadruple (Participant, Care Provider, Investigator, Outcomes Assessor)<br><br>• Primary Purpose: Treatment	• Enrollment: 60<br><br>• Ages: 40 to 80 years<br><br>• Gender: All<br><br>• Inclusion: 1. 40-80 years of age.<br><br>2.Chronic HF with reduced EF (left ventricle EF 25-35%) and NYHA class II-III symptoms.<br><br>3.Systolic BP 110-160 mm Hg<br><br>4.Optimal standard HF therapy according to European Society of Cardiology (ESC) guidelines at a stable dose for at least 4 weeks before the screening visit.<br><br>• Exclusion criteria: 1.Estimated glomerular filtration rate (eGFR) < 45 ml/min<br><br>2.Serum potassium > 5.2	•Intervention: Sacubitril-Valsartan 49 Mg-51 mg BID or 97 mg sacubitril/103 mg valsartan BID<br><br>•Comparator: Valsartan 80 mg BID or 160 mg BID	• Primary outcome:<br>1. Change from baseline in cardiac oxygen consumption and efficiency of cardiac work [Time Frame: baseline, at week 6]	•Study Start: July 5, 2018<br><br>•Primary Completion: September 16, 2022<br><br>•Study Completion: December 16, 2022<br><br>•First Posted: October 3, 2017<br><br>•Results First Posted: No Results Posted<br><br>•Last Update Posted: April 1, 2021	Novartis Pharmaceuticals<br><br>Funder type:<br>-<br>Industry	•Novartis Investigative Site, Turku, Finland	The study will assess the effects of 6 weeks of stable sacubitril/valsartan therapy, as compared with valsartan therapy, on cardiac oxygen consumption and the efficiency of cardiac work in patients with NYHA II-III heart failure (HF) and reduced systolic function using 11C-acetate positron emission tomography (PET) and echocardiography.

						mmol/l and creatinine >1.5 x ULN						

4	• NCT number: NCT04206501 • Title Acronym: (OPTIMED-HF) • Other Ids: 00003638	OptiVol for Precision Medical Management of Heart Failure	Recruiting	•Ischemic and Non-ischemic Cardiomyopathy	Study Type: Interventional (Clinical Trial)<br><br>Phase:<br>•Not Applicable<br><br>Study Design:<br>•Allocation: Randomized<br><br>•Intervention Model: Parallel Assignment<br><br>•Masking: None (Open Label)<br><br>•Primary Purpose: Treatment	Estimated Enrollment: 30 participants<br>Age:<br>18 Years to 85 Years (Adult, Older Adult)<br>Gender:<br>All<br><br>Inclusion Criteria:<br><br>•ICD implantation per guidelines for primary prevention in patients with ischemic and non-ischemic cardiomyopathy ≥3 calendar months ago.<br>•SMART Phone or tablet with Bluetooth capability with internet access.<br>•No other identifiable reversible cause explaining the left ventricular dysfunction (exclusion details present in the study.)	Device: Medtronic ICD with Opti Vol-Monitor	Primary Outcome:<br>•The rate of change in medical management with beta-blockers, diuretics and sacubitril/Valsartan (defined as initiation, termination, switch, or dosing adjustment) in the intervention group compared to usual care.<br><br>Secondary Outcome:<br>•Recurrent changes to medication type reflecting ongoing personalized optimization.<br>•Recurrent changes to medication dosing reflecting ongoing personalized<br>•Mean changes in functional status (measured by 6-minute walk distance) in the intervention vs. control groups.<br>•Percentage change in average daily activity level.<br>•Quality of life (measured by Kansas City Cardiomyopathy Questionnaire) in the intervention vs. control groups.<br>(and 4 more)	Study Start: February 5, 2020<br><br>Primary Completion: September 2021<br><br>Study Completion: January 2022<br><br>First Posted: December 20, 2019<br><br>Results First Posted: No Results Posted<br><br>Last Update Posted: April 6, 2021	•University of Rochester<br>•Medtronic<br><br>Funder Type:<br>•Other<br>•Industry	•University of Rochester Medical Center Rochester, New York, United States<br><br>•The Israeli Center for Cardiovascular Reseach	This clinical study is designed to show that a multidisciplinary team following a pre-specified standard of care medication decision model based on data from an implanted cardioverter device will increase the rate of change in Guideline Directed Medical Therapy (GDMT) in the intervention group compared to the conventional group in patients with ischemic and non-ischemic cardiomyopathies.

5	• NCT number: NCT05117736<br><br>•Other Study ID Numbers: ICM 2021-2942<br><br>• Title Acronym: PARACYS-RV	ARNI Versus plAcebo in Patients With Congenital sYStemic Right Ventricle Heart Failure (PARACYS-RV)	Not yet recruiting	•Systemic Right Ventricle dysfunction<br><br>•Heart Failure	• Study Type: Interventional<br><br>•Phase:Not Applicable<br><br>•Allocation: Randomized<br><br>•Intervention Model: Crossover Assignment<br><br>•Masking: Quadruple (Participant, Care Provider, Investigator, Outcomes Assessor)<br><br>•Primary Purpose: Treatment	• Enrollment: 48<br><br>• Ages: 18 Years and older<br><br>• Gender: All<br><br>Inclusion Criteria:<br>1. Age > or egal18 years with clinical follow-up at the Montreal Heart Institute Adult Congenital Heart Center<br><br>2. Systemic right ventricle (transposition of great vessels and atrial switch or congenitally corrected transposition of great vessels)<br><br>3. Moderate to severe systemic right ventricle dysfunction by transthoracic echocardiography (TTE) or right ventricle ejection fraction (RVEF)	•Intervention Drug: Sacubitril / Valsartan each patient will be randomized to active therapy (50, 100, or 200 mg bid of Sacubitril /Valsartan based on the run-in phase)<br><br>•Comparator: Drug: Placebo	• Primary outcome: [Time Frame: End of each arm treatment at 32 weeks and 58 weeks.]<br>1. Change of sub-maximal total exercise duration<br><br>2. Change of NT-proBNP level<br><br>• Secondary outcome: [Time Frame: End of each arm treatment at 32 weeks and 58 weeks.]<br>1. Change of quality of life measured by Kansas City Cardiomyopathy Questionnaire-12 Score<br><br>2. Change of number of participants with treatment-related adverse events as assessed by CTCAE v4.0	•Study Start: November 15, 2021<br><br>•Primary Completion: November 1, 2023<br><br>•Study Completion: November 12, 2024<br><br>•First Posted: November 11, 2021<br><br>•Results First Posted: No Results Posted<br><br>•Last Update Posted: November 22, 2021	• Novartis Pharmaceuticals<br><br>• Montreal Heart Institute<br><br>•Funder type: Industry, others.	N/A.	to assess the efficacy of Sacubitril/Valsartan over placebo in improving exercise capacity and neurohormonal activation in adults with moderate to severe systemic RV dysfunction and NYHA class II or III symptoms.

						<40% by MRI<br><br>4. NYHA Functional class II-III symptoms or peak exercise capacity <80% of predicted on a previous standard treadmill exercise stress test (usually done every two years in our congenital clinic).<br><br>5. Ability to provide informed consent to the study<br><br>6. Access or own a telephone and/or access to internet connection for teleconference call<br><br>7. Own a mailing address to receive the medication by post (FedEx or Dicom)<br><br>8. Able to perform self-measurement of the blood pressure						

						using Upper Arm Digital Blood Pressure Monitor as recommended by Hypertension Canada.<br><br>(exclusion details present in the study.)						

6	• NCT number:NCT04107220<br><br>•Other Study ID Numbers : CAN0014	Comparison of BNP and NT-proBNP in the Management of Patients With Chronic and Acute Heart Failure	Unknown status	•Heart Failure	Study Type: Interventional<br><br>•Phase: Not Applicable<br><br>•Allocation: N/A<br><br>•Intervention Model: Single Group Assignment<br><br>•Masking: None (Open Label)<br><br>•Primary Purpose: Treatment	•Enrollment : 300<br><br>•Age: 18 Years and older<br><br>• Gender: All<br><br>•Inclusion Criteria:<br>1. Out Patient Protocol - Patients with chronic heart failure being followed in the heart failure clinic eligible to switch angiotensin converting enzyme inhibitors (ACEI) or angiotensin receptor blockers (ARB) to ARNI (Entresto).<br><br>2. In Patient Protocol - Patients admitted due to acute decompensated heart failure<br><br>(exclusion details are not available in the study.)	•Intervention :One arm study patients<br>•Drug: Sacubitril / Valsartan	• Primary outcome:<br><br>1. Out Patient Protocol [Time Frame: Baseline, 2 weeks, 4 weeks, 6 months, and 12 months]<br><br>2.In Patient Protocol [Time Frame: Admission, discharge, 30th day post-discharge, 60th day post-discharge, and 180 days post discharge]	•Study Start: June 2016<br><br>•Primary Completion: May 2017<br><br>•Study Completion: June 2020<br><br>•First Posted: September 27, 2019<br><br>•Results First Posted: No Results Posted<br><br>•Last Update Posted: September 27, 2019	•Unity Health Toronto<br><br>•Alere, Inc.<br><br>•Funder type: Industry, others.	•St. Michael's Hospital , Toronto, Ontario, Canada	The purpose of this study is to compare the changes in B-type Natriuretic Peptide (BNP) and amino-terminal fragment of proBNP (NT-proBNP) in outpatients managed in the heart failure

7	• NCT number: NCT04107220<br><br>•Title Acronym: REVERSE-LVH<br><br>•Other Ids: 2018/2182	Role of ARNi in Ventricular Remodeling in Hypertensive LVH (REVERSE-LVH)	Recruiting	• Hypertensive Heart Disease	•Study Type: Interventional<br><br>•Phase: Phase 3<br><br>•Allocation: Randomized<br><br>•Intervention Model: Parallel Assignment<br><br>•Masking: Single (Outcomes Assessor)<br><br>•Primary Purpose: Treatment	•Enrollment: 100<br><br>•Age: 21 to 80 Years<br><br>• Gender: All<br><br>•Inclusion Criteria:<br>1. Increased left ventricular mass on cardiovascular magnetic resonance (based on established local age- and sex-specific CMR ranges)<br>2. Essential hypertension<br><br>•Exclusion Criteria:<br>1. Known secondary causes of hypertension<br>2.Previous intolerance to angiotensin receptor blockers<br>3.History of heart failure<br>4. Stage IV/V chronic renal disease (eGFR < 30ml/min/1.73m2)<br>5. Patients with serum potassium > 5.2	•Intervention: Treatment Arm Entresto (valsartan/sacubitril) 100mg once a day<br><br>•Comparator: Controlled Arm Valsartan 40mg once a day	• Primary outcome: [Time Frame: 52 weeks]<br>1. Fibrosis volume<br><br>• Secondary outcome: [Time Frame: 52 weeks]<br>1.Left ventricular mass measured on CMR<br>2. Biomarker/biochemistry	•Study Start: May 27, 2021<br><br>•Primary Completion: July 2023<br><br>•Study Completion: January 2024<br><br>•First Posted: December 2, 2020<br><br>•Results First Posted: No Results Posted<br><br>•Last Update Posted: July 13, 2021	•Yale University<br><br>•Funder type: Other	•Yale New Haven Hospital, New Haven, Connecticut, United States	they will be examining a novel therapy that has the potential to induce regression cardiac hypertrophy and fibrosis.

						mmol/L (mEg/L) at Visit 1<br>6.History of cardiovascular events (myocardial infarction, strokes and transient ischemic attacks)<br>7. Known atrial fibrillation<br>8. Being unable to understand or comply with study procedures (including CMR)<br>9. History or presence of any other disease with a life expectancy of < 3 years<br>10. Pregnant or nursing (lactating) women						

8	• NCT number: NCT04688294<br><br>• Other Id's: 3/2019	The Bio-Clinical Effects of the (Sacubitril-Valsartan) Combination on Patients With Chronic Heart Failure.	Recruiting	• Heart Failure	• Study Type: Interventional<br><br>• Phase: Phase 4<br><br>• Allocation: Non-Randomized<br><br>• Intervention Model: Parallel Assignment<br><br>• Masking: None (Open Label)<br><br>• Primary Purpose: Treatment	• Enrollment: 60<br><br>• Age: 40 Years to 60 Years (Adult)<br><br>• Sex: All<br><br>• Inclusion criteria:<br>1. Age 40 - 60 years.<br><br>2. Patients with chronic congestive heart failure class (II-IV) symptoms according to (NYHA) classification.<br><br>3. Left Ventricular Ejection Fraction of 40% or less.<br><br>4. NT-proBNP level of at least ≥400 pg/mL.<br><br>5. ACEI or ARB therapy with stable dose for prior 4 weeks, equivalent to enalapril ≥ 10 mg/day.<br><br>(exclusion details present in the study.)	• Drug: Sacubitril - Valsartan	• Primary outcome:<br>1. change in NTproBNP plasma concentration [Time Frame: at baseline]<br>2. change in NTproBNP plasma concentration [Time Frame: at 6 months]<br>3. severity of congestive heart failure. [Time Frame: at baseline]<br>4. severity of congestive heart failure. [Time Frame: at 6 months]<br><br>• Secondary outcome: [Time Frame: every month, up to 6 months]<br>1. change in plasma potassium concentration.<br>2. change in serum sodium concentration.<br>3. change in serum creatinine.<br><br>(more details in the study.)	• Study Start: January 1, 2021<br><br>• Primary Completion: May 2021<br><br>• Study Completion: June 2021<br><br>•First Posted: December 29, 2020<br><br>•Results First Posted: No Results Posted<br><br>•Last Update Posted: April 14, 2021	• Amir Safwat<br><br>• Suez Canal University<br><br>• Funder type: Other	Wadi El-Neel Hospital, Cairo, Egypt	Evaluate the bio-clinical effects of Entresto in the treatment of congestive heart failure patients.

9	• NCT number: NCT05021419<br><br>• Other IDs: Qehkl<br><br>• Title Acronym: SHORT	Efficacy of a Streamlined Heart Failure Optimization Protocol	Not yet recruiting	• Heart Failure With Reduced Ejection Fraction<br><br>• Chronic Heart Failure	• Study Type: Interventional<br><br>• Phase: Not Applicable<br><br>• Allocation: Randomized<br><br>• Intervention Model: Parallel Assignment<br><br>• Masking: None (Open Label)<br><br>• Primary Purpose: Treatment	• Enrollment: 60<br><br>• Ages: 18 Years and older<br><br>• Gender: All<br><br>• Inclusion:<br>1. Ejection fraction of less than 35%<br><br>2. Increased NT-pro BNP levels > 600 pg per milliliter or ≥400 pg per milliliter if they had been hospitalized for heart failure within the previous 12 months.<br><br>(exclusion details present in the study.)	•Intervention: Streamlined protocol arm<br><br>•Comparator: Standard Arm	• Primary outcome:<br>1. Time to point of optimization [Time Frame: Maximum follow-up 6 months]<br><br>• Secondary outcome: [Time Frame: Maximum follow-up 6 months]<br>1. Degree of optimization reached<br>2. Number of appointments required<br>3. Number of Complications.<br>4. Change in NT-pro BNP<br>5. Symptomatic change<br>6.Composite of cardiovascular death and worsening heart failure<br><br>(more details in the study.)	• Study start: August 2021<br><br>• Primary Completion: August 2022<br><br>• Study Completion: February 2023<br><br>• First Posted: August 25, 2021<br><br>• Results First Posted: No Results Posted<br><br>• Last Update Posted: August 25, 2021	• The Queen Elizabeth Hospital King's Lynn NHS Foundation Trust<br><br>• Novartis Pharmaceuticals<br><br>•Funder Type: Other	• Queen Elizabeth Hospital King's Lynn, King's Lynn, Norfolk, United Kingdom	

10	• NCT number: NCT04872959<br><br>• Other IDs: Pro00047392<br><br>• Title Acronym: TRANSFORMHFrEF	TRANSFORM Heart Failure With Reduced Ejection Fraction	Not yet recruiting	• Heart Failure, Systolic	• Study Type: Interventional<br><br>• Phase: Not Applicable<br><br>• Allocation: Randomized<br><br>• Intervention Model: Parallel Assignment<br><br>• Masking: Double (Participant, Care Provider)<br><br>• Primary Purpose: Treatment	• Enrollment: 3072<br><br>• Ages: 18 Years and older<br><br>• Gender: All<br><br>• Inclusion:<br>- Age ≥18 years.<br><br>- Clinical diagnosis of HF with EF ≤40% documented within 1 year of enrollment<br><br>- Receiving ≥1 oral medication for HF at study enrollment (including diuretics, ACEI/ARB/ARNI, beta-blockers, MRA, SGLT2i or thiazide diuretics.<br><br>(exclusion details present in the study.)	•Intervention: Interventional-Health Reveal<br><br>•Comparator: Usual Care Arm	• Primary outcome:<br>1. Average composite endpoint of target dose achievement for the main classes of drugs (RASi/beta blockers/MRA/SGLT2i) among eligible patients without documented contraindications or intolerance. [Time Frame: baseline to 6 months]<br><br>• Secondary outcome:<br>1. Relative change in actual achieved doses of individual classes of pivotal therapies (RASi/beta blocker/ MRA/SGLT2i). [Time Frame: baseline to 6 months]<br><br>2. Change in achievement of target doses [Time Frame: baseline to 6 months]<br><br>(more details in the study.)	• Study start: October 2021<br><br>• Primary Completion: January 2023<br><br>• Study Completion: May 2024<br><br>• First Posted: May 5, 2021<br><br>• Results First Posted: No Results Posted<br><br>• Last Update Posted: September 5, 2021	• Massachusetts General Hospital<br>.<br>• American College of Cardiology<br><br>• Funder Type: Other	• Not posted yet	

11	• NCT number: NCT03608085<br><br>• Other IDs: 1087691-2	Community Pharmacy Medication Therapy Management for Heart Failure	Recruiting	• Heart Failure	• Study Type: Interventional<br><br>• Phase: Not Applicable<br><br>• Allocation: Non-Randomized<br><br>• Intervention Model: Single Group Assignment<br><br>• Masking: None (Open Label)<br><br>• Primary Purpose: Health Services Research	• Enrollment: 83<br><br>• Ages: 18 Years and older<br><br>• Gender: All<br><br>• Inclusion:<br>- All licensed pharmacists aged ≥18 years that are employed at least part time (minimum of 4 hours per week) in a community pharmacy located in either Newport or Bristol Counties.- Implanted with an ICD or CRT-D within 2 weeks<br>- All licensed pharmacists who anticipate working in a community pharmacy located in either Newport or Bristol Counties for the next 6 months<br>- Able to sign informed consent The State of Rhode	•Intervention: Pharmacist Heart failure MTM training<br><br>•Comparator: Patient Heart failure MTM intervention	• Primary outcome:<br>1. Change in the 31-item MTM self-efficacy scale [Time Frame: baseline to 180 days]<br><br>• Secondary outcome:<br>1.Change in the Conditions of Work Effectiveness Questionnaire II (CWEQ-II Scale) [Time Frame: baseline to 180 days]<br><br>(more details in the study.)	• Study start: May 23, 2018<br><br>• Primary Completion: January 1, 2023<br><br>• Study Completion: January 1, 2023<br><br>• First Posted: July 31, 2018<br><br>• Results First Posted: No Results Posted<br><br>• Last Update Posted: October 11, 2021	• Providence VA Medical Center<br>• Lifespan<br>• University of Rhode Island<br><br>• Funder Type: U.S. Fed and Other	• Ocean State Research Institute, Providence, Rhode Island, United States<br><br>• Providence VAMC, Providence, Rhode Island, United States	

						Island pharmacy licensure requirements are as follows:<br>- Completion of a first professional degree program in pharmacy located within the United States and accredited by the American Council on Pharmaceutical Education.<br>- Completion of 1,500 internship hours.<br>- Passage of the North American Pharmacist Licensure Examination (NAPLEX), administered through the National Association of Boards of Pharmacy.<br>- Passage of the Multistate Pharmacy Jurisprudence Examination (MPJE) for Rhode Island, administered through the National Association						

						of Boards of Pharmacy. (exclusion details present in the study.)						

12	• NCT number: NCT03821701<br><br>•Other Ids: XJTU1AF-CRF-2018-019	Effect of Angiotensin-Neprilysin Inhibition (ARNI) on Prognosis of Chronic Heart Failure	Recruiting	•Chronic Heart Failure	•study Type: Interventional<br><br>•Phase:Not Applicable<br><br>•Allocation: Randomized<br><br>•Intervention Model: Parallel Assignment<br><br>•Masking: None (Open Label)<br><br>•Primary Purpose: Treatment	•Enrollment: 340<br><br>•Age: 18 Years and older<br><br>• Gender: All<br><br>•Inclusion Criteria:<br>1. Age ≥ 18<br>2. HFrEF, defined as LVEF≤40% and New York Heart Association (NYHA) class ≥ II.<br>3. Plasma NT-proBNP ≥ 600 pg/ml, or NT-proBNP ≥ 400 pg/ml if patients have been hospitalized for heart failure in 12 months.<br>4.If patients have been taking ACEI/ARB at recruitment, a stable dose equivalent to at least 10mg/day of enalapril will be required.<br>5. Volunteer for the study and sign the informed consent.<br><br>Exclusion Criteria:<br>1.Symptom	•Intervention:Drug: Entresto Sacubitril Valsartan Sodium Tablets, 100mg bid.<br><br>•Comparator:Drug: ACEI/ARB Choose one of ACEI/ARB according to the clinical condition among the whole study.	• Primary outcome:<br>1.Cardiovascular events [Time Frame: From date of randomization until the date of first documented progression or date of death from any cause, whichever came first, assessed up to 12 months]<br><br>• Secondary outcome: [Time Frame: Test in 1, 3, 6, 12 months comparing to the baseline.]<br>1.Six-minutes walking test<br>2.Left ventricular ejection fraction LVEF	•Study Start: June 1, 2018<br><br>•Primary Completion: January 31, 2021<br><br>•Study Completion: January 31, 2022<br><br>•First Posted: January 30, 2019<br><br>•Results First Posted:<br>•No Results Posted<br><br>Last Update Posted: August 7, 2019	•First Affiliated Hospital Xi'an Jiaotong University<br>•Baoji Central Hospital<br>•First Affiliated Hospital Xi'an Medical University<br><br>•Funder type: Other	•First Affiliated Hospital of Xi'an Jiaotong University, Xi'an, Shaanxi, China	they apply this trial to find if ARNI could replace ACEI/ARB to provide insights for better treatment of chronic heart failure in China.

						atic hypotension, systolic blood pressure < 95 mmHg at baseline.<br>2. eGFR < 30 ml(/min*1.73m2) at baseline.<br>3.Serum potassium > 5.4 mmol/L at baseline.<br>4. Contraindication of ACEI or ARB.						

13	• NCT number: NCT04235062 • Other Id's: CHF201901 • Title Acronym: NATRIUM-HF	NATriuretic Response to Expansion and dIUretics in huMans With Heart Failure	Not yet recruiting	•Heart Failure	Study Type: Interventional.<br><br>Phase: Not Applicable.<br><br>•Allocation: N/A<br><br>•Intervention Model: Single Group Assignment<br><br>•Masking: None (Open Label)<br><br>•Primary Purpose: Basic Science.	•Enrollment: 230 participants.<br><br>•Age: 18 Years to 79 Years (Adult, Older Adult).<br><br>•Gender: All.<br><br>Inclusion Criteria:<br><br>•Age ≥18 to <80 years.<br>•Ambulatory patients with a diagnosis of stable NYHA class II heart failure with left ventricular ejection fraction <40%, for whom sacubitril/valsartan (Entresto®) is indicated as recommended by ESC guidelines and are about to be switched to Entresto® therapy.<br>•Stable oral doses of ACEi or ARB, beta-blocker, mineralocorticoid antagonist (MRA), and loop	•Drug: Ringer's (8.6 g/L sodium chloride, 0.33 g/L calcium chloride, 0.3 g/L potassium chloride) Solution<br><br>•Drug: Intravenous Bolus Furosemide	Primary Outcome:<br>•Natriuretic response to intravascular volume expansion.<br>•Natriuretic response to IV diuretic administration.<br><br>Secondary Outcome:<br>•Natriuretic peptide (NP) response to intravascular volume expansion<br>•Natriuretic peptide response to IV diuretic administration<br>•Diuretic response<br>•Changes in dyspnea as measured by a 1-10 scale in response to intravascular fluid expansion, and diuretic administration	Study Start: April 1, 2020.<br><br>Primary Completion: July 31, 2021.<br><br>Study Completion: July 31, 2021.<br><br>First Posted: January 21, 2020.<br><br>Results First Posted: No Results Posted.<br><br>Last Update Posted: February 13, 2020.	•Momentum Research, Inc.<br><br>•Saint-Louis-Lariboisière University Hospitals<br><br>•Abbott<br><br>Funder Type:<br>•Industry<br>•Other		NATRIUM-HF is a multicenter, non-randomized, pre-post intervention study designed to assess renal response to intravascular fluid expansion and diuretics after sacubitril/valsartan (Entresto®) in euvolemic heart failure patients with reduced ejection fraction.

						diuretic (up to 120 mg daily furosemide or equivalent*) for > 3 months prior to Screening, except where intolerance or contraindication documented.<br>• And more.							

14	• NCT number: NCT05089539 • Other Id's: 2021-10-21 • Title Acronym: ARNICFH	The Effect of Angiotensin Receptor-Neprilysin Inhibition on Cardiac Fibrosis in Patients With HFpEF	Not yet recruiting	•Heart Failure With Preserved Ejection Fraction	Study Type: Interventional<br><br>Phase: Phase 2<br><br>Study Design:<br>•Allocation: Randomized<br><br>•Intervention Model: Parallel Assignment<br><br>•Masking: None (Open Label)<br><br>•Primary Purpose: Treatment	Enrollment: 60 participant<br><br>Age: 45 Years and older (Adult, Older Adult)<br><br>Gender: All<br><br>Inclusion Criteria:<br><br>•Signed and dated written informed consent<br>•Age ≥ 45 years at time of screening<br>•Preserved systolic left ventricular function, defined by left ventricular ejection fraction (LVEF) ≥ 50%<br>•NYHA classes II-IV<br>•H2FPEF score ≥ 6 or HFA-PEFF score ≥ 5 (exclusion details present in the study.)	•Drug Intervention: Angiotensin Receptor-Neprilysin Inhibition<br><br>•Comparator: Placebo	Primary Outcome Measures :<br>•Extracellular volume [ECV]<br><br>Secondary Outcome Measures :<br>•myocardial infarction, hospitalization for heart failure and death	Study Start: November 1, 2021<br><br>Primary Completion: February 1, 2022<br><br>Study Completion: April 1, 2022<br><br>First Posted: October 22, 2021<br><br>Results First Posted: No Results Posted<br><br>Last Update Posted: October 22, 2021	•Chongqing Medical University<br><br>Funder Type:<br>•Other	•The first affiliated Hospital of Chongqing Medical University, Chongqing, China	The PARAGON-HF trial conducts a prospective randomized controlled trial to evaluate the effect of ARNI on cardiac fibrosis in patients with HFpEF by cardiac magnetic resonance (CMR).

15	• NCT number: NCT03928158<br><br>• Other Ids: AAAA-A18-118022290061-2	LCZ696 in Advanced LV Hypertrophy and HFpEF	Recruiting	•Heart Failure<br><br>•Essential Hypertension	Study Type: Interventional (Clinical Trial)<br><br>Phase: Phase 2<br><br>Study Design:<br>•Allocation: Randomized<br><br>•Intervention Model: Parallel Assignment<br><br>•Masking: None (Open Label)<br><br>•Primary Purpose: Treatment	Estimated Enrollment: 60 participants<br><br>Age:<br>40 Years to 80 Years (Adult, Older Adult)<br><br>Gender:<br>All<br><br>Inclusion Criteria:<br>•Moderate/severe hypertensive left ventricular (LV) hypertrophy (LVMi ≥109 g/m² in women and ≥132 g/m² in men);<br>•New York Heart Association (NYHA) class II-III heart failure; Left ventricular ejection fraction > 50%;<br>•Increased LV filling pressures assessed at rest or at peak exercise by echocardiography<br>•Body mass index (BMI) > 30 kg/m²<br>•Signed and data informed consent (exclusion	•Drug: LCZ 696<br><br>•Drug: Valsartan	Primary Outcome Measures :<br>•Change in 6-minute walking distance (6MWD)<br><br>Secondary Outcome Measures :<br>•Change in exercise time during diastolic stress-test (DST)<br>•Change in left atrial volume index (LAVI)<br>•Change in average E/e' ratio<br>•Change estimated pulmonary artery systolic pressure (PASP)<br>•Change in left ventricular mass index (LVMI)<br>•Change of New York Heart Association (NYHA) functional classification<br>(and 10 more)	Study Start: May 31, 2019<br><br>Primary Completion: November 2020<br><br>Study Completion: November 2020<br><br>First Posted: April 26, 2019<br><br>Results First Posted: No Results Posted<br><br>Last Update Posted: December 23, 2019	•National Medical Research Center for Cardiology, Ministry of Health of Russian Federation<br><br>•Ministry of Health of Russian Federation<br><br>Funder Type:<br>•Other	•National Medical Research Center for Cardiology, Moscow, Russian Federation	Patients with advanced LVH and HFpEF will be randomly assigned in open-label fashion to receive LCZ696 titrated to 200 mg twice daily or valsartan titrated to 160 mg twice daily, and will be treated for 24 weeks.

						details present in the study.)						

16	• NCT number: NCT04164732<br><br>• Other Ids: CLCZ696I12201<br>2019-003098-24	Study of Efficacy of Oral Sacubitril/ Valsartan in Adult Patients With Non-obstructive Hypertrophic Cardiomyopathy	Recruiting	•Cardiomyopathy, Hypertrophic	Study Type: Interventional<br><br>Phase: Phase 2<br><br>Study Design:<br>•Allocation: Randomized<br><br>•Intervention Model: Parallel Assignment<br><br>•Masking: Double (Participant, Investigator)<br><br>•Primary Purpose: Treatment	Enrollment: 44 Participants<br>Age: 18 Years and older (Adult, Older Adult)<br>Gender: All<br><br>Inclusion Criteria:<br>•Diagnosed with Hypertrophic Cardiomyopathy with a left ventricular wall thickness greater than or equal to 13mm as determined by the echocardiogram obtained during the screening/baseline period<br>•Left ventricular ejection fraction (LVEF) greater than or equal to 50% as determined by echocardiogram obtained during the screening/baseline period<br>•and a few more (exclusion details	•Drug: LCZ696<br><br>•Drug: Placebo	Primary Outcome:<br>•Change from baseline in peak VO2 as measured by cardiopulmonary exercise test (CPET)	Study Start: January 8, 2020<br><br>Primary Completion: May 4, 2023<br><br>Study Completion: May 4, 2023<br><br>First Posted: November 15, 2019<br><br>Results First Posted: No Results Posted<br><br>Last Update Posted: October 18, 2021	•Novartis Pharmaceuticals<br><br>Funder Type:<br>•Industry	•Novartis Investigative Site, Stanford, California, United States<br>•Novartis Investigative Site, Boston, Massachusetts, United States<br>•Novartis Investigative Site, Boston, Massachusetts, United States<br>•Novartis Investigative Site, Boston, Massachusetts, United States<br>•Novartis Investigative Site, Ann Arbor, Michigan, United States<br>•Novartis Investigative Site, Morristo	The purpose of this study is to determine if LCZ696 is safe, tolerable and can improve exercise capacity (via improved peak VO2) in non-obstructive HCM patient population over the course of 50 weeks of treatment.

						present in the study.)					wn, New Jersey, United States<br>•Novartis Investigative Site, Portland, Oregon, United States<br>•Novartis Investigative Site, Tampere, Finland<br>•Novartis Investigative Site, Berlin, Germany<br>•Novartis Investigative Site, Berlin, Germany<br>•and 13 more	

17	• NCT number: NCT03832660<br><br>• Title Acronym: SILICOFCM<br><br>• Other Ids: 252015	Sacubitril/ Valsartan vs Lifestyle in Hypertrophic Cardiomyopathy	Recruiting	•Hypertrophic Cardiomyopathy	Study Type: Interventional<br><br>Phase: Phase 2<br><br>Study Design:<br>•Allocation: Randomized<br><br>•Intervention Model: Parallel Assignment<br><br>•Masking: None (Open Label)<br><br>•Primary Purpose: Treatment	Enrollment: 240 Participants<br><br>Age: 18 Years to 70 Years (Adult, Older Adult)<br><br>Gender: All<br><br>Inclusion Criteria:<br>•Confirmed diagnosis of obstructive and/or non-obstructive hypertrophic cardiomyopathy.<br>•Agreement to be a participant in the study protocol and willing/able to return for follow-up. (exclusion details present in the study.)	•Behavioral: Lifestyle<br><br>•Drug: Sacubitril / Valsartan	Primary Outcome Measures :<br>•Peak Oxygen Consumption (ml/kg/min)<br><br>Secondary Outcome Measures :<br>•Left ventricular mass (grams)<br>•LVOT obstruction<br>•LVEF (%)<br>•Minnesota Living with Heart Failure questionnaire<br>•SF36 questionnaire<br>•E/A ratio	Study Start: May 3, 2019<br><br>Primary Completion: February 28, 2022<br><br>Study Completion: June 30, 2022<br><br>First Posted: February 6, 2019<br><br>Results First Posted: No Results Posted<br><br>Last Update Posted: September 29, 2021	•Newcastle University<br><br>•Azienda Ospedaliero Universitaria Careggi<br><br>•University Hospital Regensburg<br><br>•Institute for Cardiovascular Diseases of Vojvodina<br><br>•University of Belgrade<br><br>Funder Type:<br>•Other	•University Hospital Regensburg, Regensburg, Germany<br>•Azienda Ospedaliero Universitaria Careggi Florence, Florence, Italy<br>•University of Belgrade Faculty of Medicine, Belgrade, Serbia<br>•Institute of Cardiovascular Diseases of Vojvodina, Sremska Kamenica, Serbia<br>•Newcastle University, Faculty of Medical Sciences, Clinical Research Facility, Royal	The overall aim of this project is to establish potential benefits of a novel lifestyle (physical activity and dietary nitrate) and pharmacological (angiotensin receptor neprilysin inhibitor) interventions in patients with hypertrophic cardiomyopathy (HCM).

											Victoria Infirmary; Newcastle upon Tyne Hospitals NHS Foundation Trust, Newcastle Upon Tyne, England, United Kingdom	

18	• NCT number: NCT04687111<br><br>•Title Acronym: PARABLE<br><br>•Other Ids:<br>• HBT-PTCL-01, SVUH-2015-002<br>• 2015-002928-53	Personalised Prospective Comparison of ARni With ArB in Patients With Natriuretic Peptide eLEvation (PARABLE)	Active, not recruiting	•Atrial Remodeling<br>• Myocardial Dysfunction<br>•Left Ventricular Remodeling<br>•Left Ventricular Diastolic Dysfunction<br>• Hypertension<br>• Cardiovascular Morbidity<br>•Fibrosis Myocardial<br>• Inflammatory Myopathy<br>•Atrial Arrhythmia	•Study Type: Interventional<br><br>•Phase: Phase 2<br><br>•Allocation: Randomized<br><br>•Intervention Model: Parallel Assignment<br><br>•Masking: Quadruple (Participant, Care Provider, Investigator, Outcomes Assessor)<br><br>•Primary Purpose: Prevention	•Enrollment : 250<br><br>•Age:40 Years and older<br><br>• Gender: All<br><br>•Inclusion Criteria:<br><br>1. Age > 40yrs with cardiovascular risk factor(s) including at least one of:<br>A. History of hypertension (medicated for greater than one month);<br>B. History of diabetes;<br>2. Elevated NP: Elevated NP: BNP between 20 and 280pg/ml or NT-proBNP values between 100 pg/ml and 1,000 pg/ml within 6 months prior to screening or at screening<br>3. LAVI > 28 mL/m2 obtained during Doppler Echocardiography within 6 months	•Experimental: Intervention Sacubitril/Valsartan 50mg bid titrated to maximum dose of 200mg bid<br><br>•Active Comparator: Control Valsartan 40mg bid titrated to maximum dose of 160mg bid	• Primary outcome:<br>1.Change in Left Atrial Volume Index (LAV/BSA*) [Time Frame: Baseline-18 months]<br><br>• Secondary outcome:[Time Frame: Baseline -18 months]<br>1.Change in left ventricular function (E/e')<br>2.Change in left atrial volume index (LAV/BSA*)<br>3.Change in left atrial function measured as total left atrial ejection fraction (LAEF)<br>4.Change in left atrial function measured as left atrial stroke volume index<br>5. Change in left ventricular structure measured as LVMi<br>6. Change in left ventricular function (LVEF)<br>7.Change in measures of vascular compliance (pulse pressure)<br>8. Change in natriuretic peptide biomarker profile<br>9. Time to first all cardiovascular death and major adverse cardiac events (MACE) requiring hospitalisation over 18 months<br>10. Change in Left Atrial Volume Index (LAVi) analysed per protocol.	•Study Start: December 16, 2015<br><br>Primary Completion: June 11, 2021<br><br>•Study Completion: June 11, 2021<br><br>•First Posted: December 29, 2020<br>•Results First Posted: No Results Posted<br><br>•Last Update Posted: May 11, 2021	•Mark Ledwidge<br>•The Heartbeat Trust<br>•St Vincent's University Hospital, Ireland<br><br>•Funder type: Other	•The STOP-HF Service, St Michael's Hosptial, Dun Laoghaire, Co Dublin, Ireland<br>•St Vincents University Hospital, Dublin, Ireland	The PARABLE study investigates the hypothesis that sacubitril-valsartan can provide benefits in terms of left atrial structure and function as well as left ventricular structure and function in asymptomatic (stage A/B HFpEF) patients.

						prior to screening or at screening<br>4. Subjects must give written informed consent to participate in the study and before any study related assessments are performed.						
Advanced Heart Failure												

1	• NCT number: NCT04103554 • Other Id's: ENVAD-HF<br>• Title Acronym: ENVAD-HF	Sacubitril/ Valsartan in Left Ventricular Assist Device Recipients	Recruiting	• Heart Failure	• Study Type: Interventional<br><br>• Phase: Phase 4<br><br>• Allocation: Randomized<br><br>• Intervention Model: Parallel Assignment<br><br>• Masking: None (Open Label)<br><br>• Primary Purpose: Treatment	• Enrollment: 60<br><br>• Age: 18 Years to 90 Years (Adult, Older Adult)<br><br>• Sex: All<br><br>• Inclusion criteria:<br>1. signed informed consent.<br><br>2. ≥18 years of age, male or female.<br><br>3. Recently implanted HeartMate 3 LVAD recipients, in stable condition and ready for discharge or chronic, stable, ambulatory HeartMate 3 LVAD carriers implanted within 1 year prior to enrolment.<br><br>(exclusion details present in the study.)	• Drug: Sacubitril-Valsartan<br><br>• Drug: Standard of care	• Primary outcome: [Time Frame: 3 months]<br>1. Freedom from all-cause death, deterioration in renal function (reaching end-stage renal disease (ESRD), renal death or 50% decline in eGFR), hyperkalemia or symptomatic hypotension.<br><br>• Secondary outcome:<br>1. Change in NT-proBNP from enrolment to 8 weeks [Time Frame: 8 weeks]<br>2. Change in Burden of hemocompatibility (hemocompatibility score) [Time Frame: 3 months, 12 months]<br>3. Number of RV failure events [Time Frame: 3 months, 12 months]<br>4. Time to first unplanned hospitalisation [Time Frame: 3 months, 12 months]<br>5. Number of unplanned hospitalizations [Time Frame: 3 months, 12 months]<br>6. Change in blood-pressure lowering medications [Time Frame: 3 months, 12 months]<br>7. Change in eGFR values [Time Frame: 3 months, 12 months]<br><br>(more details in the study.)	• Study Start: February 5, 2021<br><br>• Primary Completion: November 2022<br><br>• Study Completion: August 2023<br><br>• First Posted: September 25, 2019<br><br>• Results First Posted: No Results Posted<br><br>• Last Update Posted: April 29, 2021	• University of Zagreb<br><br>• Funder type: Other	University Hospital Centre Zagreb, Zagreb, Croatia	The study asesses the use of Sacubitril/Valsartan in HeartMate 3 LVAD patients.

2	• NCT number: NCT04191681 • Other Id's: 19-1132 • Title Acronym: SEAL-IT	Safety and Efficacy of ARNI After LVAD ImplanT (SEAL-IT) Study	Recruiting	• Stage D Heart Failure	• Study Type: Interventional<br><br>• Phase: Phase 4<br><br>• Allocation: Randomized<br><br>• Intervention Model: Parallel Assignment<br><br>• Masking: None (Open Label)<br><br>• Primary Purpose: Treatment	• Enrollment: 50<br><br>• Age: 18 Years and older (Adult, Older Adult)<br><br>• Sex: All<br><br>• Inclusion criteria:<br>1. signed informed consent.<br><br>2. Adults (age ≥ 18 years).<br><br>3. Durable CF-LVAD for any indication.<br><br>4. NYHA II to IV classification.<br><br>5. LVEF < 40%.<br><br>(exclusion details present in the study.)	• Drug: Sacubitril-Valsartan<br><br>• Drug: Usual care (standard-of-care)	• Primary outcome: [Time Frame: 3 months]<br>1. Incidence of drug discontinuation from drug-related adverse events due to sacubitril-valsartan versus standard-of-care oral vasodilator therapy.<br>2. Time-averaged proportional change in NT-proBNP concentration (pg/mL) with sacubitril-valsartan versus standard-of-care oral vasodilator therapy.<br><br>• Secondary outcome:<br>1. Time-averaged proportional change in NT-proBNP concentration (pg/mL) with sacubitril-valsartan versus standard-of-care oral vasodilator therapy at 6 and 12 months.<br>2. Proportion of patients (%) with effective MAP control (65 to 85 mm Hg) with sacubitril-valsartan versus standard-of-care oral vasodilator therapy at 3, 6 and 12 months.<br>3. Proportion of patients (%) on other cardiac medications with sacubitril-valsartan versus standard-of-care oral vasodilator therapy at 3, 6 and 12 months relative to baseline.<br>4. Proportion of patients (%) in each New York Heart Association (NYHA) classification with sacubitril-valsartan versus standard-of-care oral vasodilator therapy at 3, 6 and 12 months relative to baseline.<br>5. Proportion of patients (%) with heart failure readmissions with sacubitril-valsartan versus standard-of-care oral vasodilator therapy at 6 and 12 months.<br>6. Absolute change in mean left ventricular end-diastolic dimension (mm) on echocardiogram with sacubitril-valsartan versus standard-of-care oral vasodilator therapy at 6 weeks and/or 3, 6 and/or 12 months.<br>7. Absolute change in mitral E/A ratio on echocardiogram with sacubitril-valsartan versus standard-of-care oral vasodilator therapy at 6 weeks and/or 3, 6 and/or 12 months.<br>8. Absolute change in indexed left atrial/right atrial volume (mL/m2) on echocardiogram with sacubitril-valsartan versus standard-of-care oral vasodilator therapy at 6 weeks and/or 3, 6 and/or 12 months.<br>9. Absolute change in mean right atrial pressure/pulmonary artery pressure (mm Hg) with sacubitril-valsartan versus standard-of-care oral vasodilator therapy at 6 weeks and/or 3, 6 and/or 12 months.<br>10. Absolute change in mean pulmonary capillary wedge pressure (mm Hg) with sacubitril-valsartan versus standard-of-care oral vasodilator therapy at 6 weeks and/or 3, 6 and/or 12 months.<br>11. Absolute change in pulmonary artery pulsatility index with sacubitril-valsartan versus standard-of-care oral vasodilator therapy at 6 weeks and/or 3, 6 and/or 12 months.<br>12. Absolute change in pulmonary vascular	• Study Start: November 8, 2019<br><br>• Primary Completion: March 2022<br><br>• Study Completion: December 2022<br><br>• First Posted: December 10, 2019<br><br>• Results First Posted: No Results Posted<br><br>• Last Update Posted: June 7, 2021	• The Cleveland Clinic<br><br>• Funder type: Other	Cleveland Clinic, Cleveland, Ohio, United States	the study investigates the safety and efficacy of sacubitril-valsartan in (CF-LVAD) implantation patients compared to the standard of care oral vasodilator therapy.

								resistance/systemic vascular resistance (Woods Unit) with sacubitril-valsartan versus standard-of-care oral vasodilator therapy at 6 weeks and/or 3, 6 and/or 12 months.<br>13. Absolute change in cardiac index (L/min/m2) with sacubitril-valsartan versus standard-of-care oral vasodilator therapy at 6 weeks and/or 3, 6 and/or 12 months.<br><br>(more details in the study.)				
Hypertension												

1	• NCT number: NCT04971720 • IRB number: IRB-300007789	Natriuretic Peptide-Renin-Angiotensin-Aldosterone System Rhythm Axis and Nocturnal Blood Pressure	Not yet recruiting	•Obesity<br>•Cardiovascular Diseases<br>•Hypertension<br>•Nocturnal Blood Pressure<br>•Natriuretic Peptides<br>•Renin-Angiotensin-Aldosterone System	• Study Type: Interventional<br><br>• Phase: Phase 2 Phase 3<br><br>• Allocation: Randomized<br><br>• Intervention Model: Factorial Assignment<br><br>• Masking: Quadruple (Participant, Care Provider, Investigator, Outcomes Assessor)<br><br>• Primary Purpose: Other	• Enrollment: 160<br><br>• Ages: 18 Years and older<br><br>• Gender: All<br><br>• Inclusion:<br>1. Age ≥18 years of age<br><br>2. Body Mass Index between 30 to 45 kg/m^2<br><br>3. Blood pressure: Systolic BP ≥ 140-160 mmHg and diastolic blood pressure ≥ 80-100mmHg.<br><br>(exclusion details present in the study.)	•Intervention: Sacubitril-Valsartan 49 Mg-51 mg Oral Tablet Standardized Study Diet<br><br>•Comparator: Valsartan 80 mg Oral Tablet Standardized Study Diet	• Primary outcome: [Time Frame: Baseline - after 7 days of intervention.]<br>1. Change in mean nocturnal systolic blood pressure<br>2. Change in mean nocturnal systolic blood pressure will be analyzed in the two study arms (morning vs. evening dose of sacubitril/valsartan or valsartan) from baseline and after 7 days of intervention.<br><br>• Secondary outcome: [Time Frame: Baseline - after 7 days of intervention.]<br>1. Change in percent nocturnal dipping blood pressure<br>2. Change in 24-hour mean blood pressure.<br>3. Change in mean daytime blood pressure.<br>4. Change in mean nocturnal diastolic blood pressure.<br>5. Change in the daytime, nocturnal, and total urinary excretion parameters (Urine Sodium, Urine Potassium, Urine Creatinine, Urine Albumin).<br>6. Change in 24-hour, daytime, and nocturnal Change in 24-hour, daytime, and nocturnal ANP, BNP, NTproBNP, and renin levels<br>7. Change in 24-hour, daytime, and nocturnal MRproANP levels<br>8. Change in 24-hour, daytime, and nocturnal Aldosterone levels<br>9. Change in rhythm parameters.<br><br>(more details in the study.)	• Study start: January 1, 2023<br><br>• Primary Completion: January 1, 2027<br><br>• Study Completion: January 1, 2027<br><br>• First Posted: July 21, 2021<br><br>• Results First Posted: No Results Posted<br><br>• Last Update Posted: July 28, 2021	•University of Alabama at Birmingham.<br><br>•Funder Type: Other	University of Alabama at Birmingham, Birmingham, Alabama, United States	If the results of this study are positive, Entresto could be used for obese patients to treat nocturnal hypertenstion.

2	• NCT number: NCT04637152 • Other Id's: HGeralRobertoSantos • Title Acronym: HEVA	Sacubitril/ Valsartan in Resistant Hypertension	Recruiting	• Resistant Hypertension<br>• Blood Pressure	• Study Type: Interventional<br><br>• Phase: Phase 2<br><br>• Allocation: Randomized<br><br>• Intervention Model: Parallel Assignment<br><br>• Masking: Single (Investigator)<br><br>• Primary Purpose: Treatment	• Enrollment: 100<br><br>• Age: 18 Years and older (Adult, Older Adult)<br><br>• Sex: All<br><br>• Inclusion criteria:<br>1. Men or women, over 18.<br><br>2. Diagnosed with resistant hypertension (using ≥ 3 antihypertensive agents of different classes - eg. ACEI, ARB, CCB, loop and thiazide diuretics or potassium-sparing diuretics), at least 4 weeks before recruitment, with a BP ≥ 140/90 mmHg.<br><br>(exclusion details present in the study.)	• Drug: Sacubitril - Valsartan	• Primary outcome: [Time Frame: 12 weeks]<br>1. Reduction in systolic blood pressure and diastolic blood pressure in the sitting position.<br>2. Mean reduction in ambulatory systolic blood pressure (maSBP) and ambulatory diastolic blood pressure.<br><br>• Secondary outcome: [Time Frame: 12 weeks]<br>1. Safety outcomes.<br><br>(more details in the study.)	• Study Start: November 11, 2020<br><br>• Primary Completion: October 11, 2022<br><br>• Study Completion: November 11, 2022<br><br>• First Posted: November 19, 2020<br><br>• Results First Posted: No Results Posted<br><br>• Last Update Posted: December 1, 2020	• Hospital Geral Roberto Santos<br>• Hospital Universitário Professor Edgard Santos<br><br>• Funder type: Other	• General Hospital Roberto Santos, Salvador, Bahia, Brazil<br><br>• Hospital Universitário Professor Edgard Santos, Salvador, Bahia, Brazil	Evaluate the efficacy and safety of sacubitril/valsartan vs optimized antihypertensive agents in resistant hypertension patients.

3	• NCT number: NCT04800081<br><br>• Other Id's: 2020A-247	Effect of LCZ696 on Urinary Microalbumin and Pulse Wave Velocity in Perimenopausal Patients With Hypertension	Recruiting	• Hypertension	• Study Type: Interventional<br><br>• Phase: Not Applicable<br><br>• Allocation: Randomized<br><br>• Intervention Model: Parallel Assignment<br><br>• Masking: None (Open Label)<br><br>• Primary Purpose: Treatment	• Enrollment: 264<br><br>• Age: 40 Years to 60 Years (Adult)<br><br>• Sex: Female<br><br>• Inclusion criteria:<br>1. Signed informed consent.<br><br>2. Patients with essential hypertension (SBP: 140-180 mmHg, and/or DBP: 90-110 mmHg) were diagnosed according to the hypertension diagnostic criteria of the Chinese Guidelines for Hypertension (2018 Revised Edition).<br><br>3. Female aged 45-55 years old and meeting the perimenopausal criteria in the STRAE+10.<br><br>4. No other complications, no treatment or ongoing	•Drug: Sacubitril / Valsartan<br><br>•Drug: Valsartan	• Primary outcome: [Time Frame: Evaluation at 12 weeks of treatment will be reported]<br>1. Blood pressure.<br>2. Urinary microalbuminuria.<br>3. Pulse wave velocity.<br>4. Ventricular mass index were measured by cardiac ultrasound.<br><br>• Secondary outcome: [Time Frame: During the drug intervention up to 30 days, 60 days and 80days, it will be reported in the final.]<br>1. Adverse events.<br><br>(more details in the study.)	• Study Start: July 9, 2020<br><br>• Primary Completion: June 9, 2021<br><br>• Study Completion: July 9, 2021<br><br>• First Posted: March 16, 2021<br><br>• Results First Posted: No Results Posted<br><br>• Last Update Posted: March 16, 2021	• LanZhou University<br><br>• Funder type: Other	• LanZhou University<br><br>• Funder type: Other	Evaluate the clinical application of Entresto to improve the blood pressure management of perimenopausal hypertension women.

						antihypertensive therapy (SBP ≥140mmHg or Diastolic BP ≥90mmHg).<br><br>(exclusion details present in the study.)						

4	• NCT number: NCT04929600 • Other Id's: PARASTRAIN	Sacubitril/ Valsartan Versus Amlodipine in Hypertension and Left Ventricular Hypertrophy.	Not yet recruiting	•Hypertension •Left Ventricular Hypertrophy	Study Type: Interventional<br><br>Phase: Phase 4<br><br>Study Design:<br><br>•Allocation: Randomized<br><br>•Intervention Model: Parallel Assignment<br><br>•Masking: Quadruple (Participant, Care Provider, Investigator, Outcomes Assessor)<br><br>•Primary Purpose: Treatment	Enrollment: 120 participant Age: 18 Years and older (Adult, Older Adult) Gender: All<br><br>Inclusion Criteria:<br>•Aged ≥18;<br>•Essential mild to moderate hypertension (office systolic blood pressure (SBP) ≥ 140 and < 180mmHg in untreated patients or patients with taking single antihypertensive drugs (not including single-pill combination).<br>•Echocardiographic diagnosis of LVH, LVMI: male≥115g/m2, female≥ 95g/m2;<br>•and more (exclusion details present in the study.)	Drug: sacubitril/valsartan<br>Drug: Amlodipine	Primary Outcome:<br>•Change in LVGLS after 24 weeks<br><br>Secondary Outcome:<br>•Change in LVGLS after 12 weeks<br>•Changes in LVMI after 24 weeks<br>•Changes in LVMI after 12 weeks<br>•Blood pressure after 24 weeks<br>•Blood pressure after 12 weeks	Study Start: June 2021<br><br>Primary Completion: June 2022<br><br>Study Completion: June 2022<br><br>First Posted: June 18, 2021<br><br>Results First Posted: No Results Posted<br><br>Last Update Posted: June 18, 2021	•Shanghai Jiao Tong University School of Medicine<br><br>Funder Type:<br>•Other	•Shanghai Institite of Hypertension, Ruijin Hospital, Shanghai Jiaotong University School of Medicine, Shanghai, China	This studt demonstrate the superior efficacy of ARNI on improvement of LVH and blood pressure control compared with amlodipine in hypertension patients with LVH.
Electrophysiology												

1	• NCT number: NCT03791723 • Title Acronym: APART-AF • Other Ids: APART-AF	Effect of ARNI in Patients With Persistent AF and Enlarged Left Atrium After Catheter Ablation	Not yet recruiting	•Atrial Fibrillation<br><br>•Cardiac Remodeling, Atrial<br><br>•Sacubitril/Valsartan	Study Type: Interventional<br><br>Phase: Not Applicable<br><br>Study Design:<br><br>•Allocation: Randomized<br><br>•Intervention Model: Parallel Assignment<br><br>•Masking: None (Open Label)<br><br>•Primary Purpose: Treatment	Enrollment: 90 participant<br><br>Age: 18 Years to 80 Years (Adult, Older Adult)<br>Gender: All<br><br>Inclusion Criteria:<br><br>•Patients with persistent atrial fibrillation undergoing catheter ablation within 2 weeks.<br>•≥18 and ≤75 years of age.<br>•Left atrium diameter（LAD）≥35mm, With or without right atrium diameter (RAD ≥40mm diagnosed by Echocardiographic.<br>•and many more. (exclusion details present in the study.)	•Drug: Sacubitril Valsartan<br>•Drug: Valsartan	Primary Outcome:<br>•Left atrial size changes compared to baseline levels<br><br>Secondary Outcome:<br>•Freedom from AF or AT without the use of antiarrhythmic drugs at 12 months after a single ablation procedure.<br><br>•all-cause death<br><br>•Time to first documented recurrence of atrial arrhythmias<br><br>•Number of hospitalizations caused by heart failure<br><br>•All-cause hospitalizations<br><br>•Number of patients requires adjustment of the drug because of Hypotension<br><br>•Change From Baseline in Echocardiography Parameters: Left Ventricular Ejection Fraction<br><br>•Right atrial size changes compared to baseline levels	Study Start: June 1, 2019<br><br>Primary Completion: January 1, 2021<br><br>Study Completion: December 30, 2021<br><br>First Posted: January 3, 2019<br><br>Results First Posted: No Results Posted<br><br>Last Update Posted: March 19, 2019	•The Second Affiliated Hospital of Chongqing Medical University<br><br>Funder Type:<br>•Other		The purpose of this clinical randomized trial is to evaluate the efficacy and safety of Sacubitril/Valsartan compared with ARB in improving cardiac remodeling in patients With Enlarged Left Atrium Diameter and Persistent AF.

2	• NCT number: NCT04491136<br><br>• Other IDs: CLCZ696BCN04	Effective Study of ARNI on Ventricular Arrhythmia in HFrEF Patients With ICD or CRT-D	Recruiting	• Heart Failure	• Study Type: Interventional<br><br>• Phase: Phase 4<br><br>• Allocation: N/A<br><br>• Intervention Model: Single Group Assignment<br><br>• Masking: None (Open Label)<br><br>• Primary Purpose: Treatment	• Enrollment: 275<br><br>• Ages: 18 Years to 80 Years<br><br>• Gender: All<br><br>• Inclusion:<br>- Male or female patients ≥18 and ≤80 years of age<br>- Implanted with an ICD or CRT-D within 2 weeks<br>- NYHA functional class II - IV<br>- LVEF ≤40% (measured by echocardiography)<br>- Signed informed consent must be obtained prior to participation in the study.<br>(exclusion details present in the study.)	•Intervention: ACEI/ARB treatment in 6 months then ARNI treatment in next 6 months<br><br>•Comparator: None	• Primary outcome:<br>1. Proportion of patients with VA, ICD and ATP events over 6 months of ACEI/ARB and 6 months of ARNI treatment [Time Frame: 12 months]<br><br>• Secondary outcome:<br>1.Numbers of NSVT, SVT, PVC, ICD shock and ATP experienced by patients [Time Frame: baseline, 12 month]<br>2.LVEF (%) [Time Frame: baseline ,12 month]<br>3. NT-proBNP (pg/mL) level [Time Frame: baseline, 12 month]<br>4. Number of hospitalizations for arrhythmia or HF related hospitalizations [Time Frame: baseline, 12 month]<br>(more details in the study.)	• Study start: November 11, 2020<br><br>• Primary Completion: August 30, 2022<br><br>• Study Completion: August 31, 2022<br><br>• First Posted: July 29, 2020<br><br>• Results First Posted: No Results Posted<br><br>• Last Update Posted: October 1, 2021	• Novartis Pharmaceuticals<br><br>• Funder Type: Other	•Novartis Investigative Site, Hefei, An Hui, China<br>•Novartis Investigative Site, Bei Jing, Bei Jing, China<br>•Novartis Investigative Site, Beijing, Beijing, China<br>•Novartis Investigative Site, Fuzhou, Fujian, China<br>•Novartis Investigative Site, Zhengzhou, Henan, China<br>•Novartis Investigative Site, Changsha, Hunan, China<br>•Novartis Investigative Site, Nanjing, Jiangsu,	

											China<br>•Novartis Investigative Site, Nanjing, Jiangsu, China<br>•Novartis Investigative Site, Nantong, Jiangsu, China<br>•Novartis Investigative Site, Wu XI, Jiangsu, China<br>•and 14 more	
Diabetes												

1	• NCT number: NCT04055428 • IRB number: IRB-300003702	NAUTICAL: Effect of Natriuretic Peptide Augmentation on Cardiometabolic Health in Black Individuals	Recruiting	• Diabetes Mellitus<br>• Cardiovascular Diseases<br>• Insulin Sensitivity/Resistance<br>• Metabolic Disease<br>• Natriuretic Peptides<br>• Metabolism<br>• Energy Expenditure	• Study Type: Interventional<br><br>• Phase: Phase 2<br><br>• Allocation: Randomized<br><br>• Intervention Model: Parallel Assignment<br><br>• Masking: Quadruple (Participant, Care Provider, Investigator, Outcomes Assessor)<br><br>• Primary Purpose: Other	• Enrollment: 200<br><br>• Age: 18 Years and older (Adult, Older Adult)<br><br>• Sex: All<br><br>• Inclusion Criteria:<br>1. Adults: Age ≥ 18 years of age<br><br>2. Race: African-American or Black<br><br>3. Blood pressure: 120-160/80-100 mmHg (either untreated or after 4 weeks of washout in those treated with up to two classes of antihypertensives)<br><br>4. Baseline insulin resistance: HOMA-IR ≥2.5<br><br>(exclusion details present in the study.)	•Drug: Sacubitril, Valsartan 97-103 mg Oral Tablet<br><br>•Drug: Valsartan 160 mg<br><br>•Other: Intravenous Glucose Tolerance Test<br><br>• Dietary Supplement: Standardized Meals<br><br>•Other: Exercise capacity VO2 maximum determination	• Primary outcome: [Time Frame: 12 weeks]<br>1. Change in insulin sensitivity after natriuretic peptide augmentation.<br>2. Change in energy expenditure after natriuretic peptide augmentation.<br><br>• Secondary outcome: [Time Frame: 12 weeks]<br>1. Change in exercise energy expenditure after 12 weeks of pharmacological intervention.<br>2. Change in post-meal increase in GLP-1 levels.<br>3. Change in peak oxygen consumption after 12 weeks of pharmacological intervention.<br>4. Change in fasting GLP-1 levels.<br>5. Change in natriuretic peptide levels.<br>6. Change in measures of insulin sensitivity.<br>7. Change in HBA1c levels.<br>8. Change in fasting blood glucose levels.<br>9. Change in HOMA-IR.<br>10. Change in fasting insulin levels.<br>11. Change in measures of body mass index.<br>12. Change in measures of hip/waist circumference.<br>13. Change in measures of adipose tissue mass.<br>14. Change in total cholesterol/LDL-C/HDL-C/triglyceride levels.<br>15. Correlation of change in MR-pro atrial natriuretic peptide levels.<br>16. Correlation of change in B-type natriuretic peptide levels.<br>17. Correlation of change in NT-pro B-type natriuretic peptide levels.<br>18. Impact of Natriuretic Peptide Genotype on Study Endpoints.<br>19. Change in Metabolomic Profile.<br><br>(more details in the study.)	• Study start: August 15, 2020<br><br>• Primary Completion: August 30, 2026<br><br>• Study Completion: August 30, 2026<br><br>• First Posted: August 13, 2019<br><br>• Results First Posted: No Results Posted<br><br>• Last Update Posted: June 1, 2021	• University of Alabama at Birmingham<br><br>• Funder type: Other	University of Alabama at Birmingham, Birmingham, Alabama, United States	The study hypothesized that the use of sacubitril/valsartan in African American individuals will have an improvement in both the insulin sensitivity and the resting & exercise energy expenditure more than with valsartan alone.
Chronic Kidney Disease												

1	• NCT number: NCT04458285 • Other Id's: 20191025 • Title Acronym: ESARHD-HF	Efficacy and Safety of Sacubitril/ Valsartan in Maintenance Hemodialysis Patients With Heart Failure	Recruiting	• Hemodialysis Complication<br>• Heart Failure	• Study Type: Interventional<br><br>• Phase: Not Applicable<br><br>• Allocation: Randomized<br><br>• Intervention Model: Parallel Assignment<br><br>• Masking: None (Open Label)<br><br>• Primary Purpose: Treatment	• Enrollment: 118<br><br>• Age: 18 Years to 80 Years (Adult, Older Adult)<br><br>• Sex: All<br><br>• Inclusion criteria:<br>1. Written informed consent.<br><br>2. End stage renal disease (ESRD) patients (eGFR<15 ml/min/1.73 m²) who have been receiving hemodialysis 3 times a week for at least 12 weeks before registration.<br><br>3. Chronic heart failure (NYHA class ≥ II) with reduced ejection fraction, defined as known LVEF ≤ 50%.<br><br>4. Mean sitting systolic blood pressure(msSBP)≥110 mmHg.<br><br>5. Use of	• Drug: Sacubitril / Valsartan Oral Tablet [Entresto]<br><br>• Drug: Valsartan 80mg Tablet	• Primary outcome: [Time Frame: 12 weeks]<br>1. Left ventricular ejection fraction (LVEF).<br><br>• Secondrary outcome: [Time Frame: 12 weeks]<br>1. N terminal pro B type natriuretic peptide (NT-prpBNP).<br>2. Left ventricular end diastolic volume (LVEDV)/Left atrial volume (LAV).<br>3. The ratio of mitral early diastolic blood flow peak and mitral annulus velocity (E/E').<br>4. Pulmonary Artery Pressure.<br>5. Concentration of high-sensitivity serum troponin T.<br>6. NYHA functional classification.<br>7. Minnesota Heart Failure Quality of Life Questionnaire (LiHFe).<br>8. Systolic and diastolic blood pressure.<br>9. Concentration of postassium.<br>10. Electrocardiogram(ECG).<br>11. Estimated glomerular filtration rate(eGFR).<br>12. Incidence of Angioedema.<br>13. Concentration of alanine aminotransferase or aspartate aminotransferase.<br>14. Neprilysin.<br><br>(more details in the study.)	• Study Start: January 1, 2020<br><br>• Primary Completion: December 31, 2020<br><br>• Study Completion: December 31, 2020<br><br>• First Posted: July 7, 2020<br><br>• Results First Posted: No Results Posted<br><br>• Last Update Posted: October 6, 2020	• Guangdong Provincial People's Hospital.<br><br>• Funder type: Other	Guangdong Provincial People's Hospital, Guangzhou, Guangdong, China	The purpose of ESARHD-HF study is to investigate the safety and the efficacy of sacubitril/valsartan in patients undergoing maintenance hemodialysis diagnosed with heart failure vs valsartan alone.

						ACEI/ARB for at least 2 weeks.<br><br>6. Good compliance.<br><br>(exclusion details present in the study.)						

2	• NCT number: NCT04572724 • Other Id's: 20200601054	The Effect of Sacubitril/ Valsartan on Cardiovascular Events in Dialysis Patients and Efficacy Prediction of Baseline LVEF Value	Recruiting	• Peritoneal Dialysis Complication<br>• Hemodialysis Complication<br>• Heart Failure	• Study Type: Interventional<br><br>• Phase: Phase 4<br><br>• Allocation: Non-Randomized<br><br>• Intervention Model: Parallel Assignment<br><br>• Masking: None (Open Label)<br><br>• Primary Purpose: Treatment	• Enrollment: 120<br><br>• Age: 18 Years to 70 Years (Adult, Older Adult)<br><br>• Sex: All<br><br>• Inclusion criteria:<br>1. written informed consent.<br><br>2. 18 to 70 years old participants, No restrictions on gender or race.<br><br>3. Stable heart failure in New York Heart Association (NYHA) class II, III, or IV symptoms, presentation of typical heart failure symptoms accompanied by HF signs caused by a structural and/or functional cardiac abnormality<br><br>4. Under maintenance dialysis (hemodialysis or peritoneal dialysis) for more than one year.	• Drug: Sacubitril/ Valsartan Oral Tablet<br><br>• Drug: RAS Inhibitors	• Primary outcome: [Time Frame: at least 18 months]<br>1. cardiovascular events.<br>2. hospitalization for heart failure.<br><br>• Secondary outcome:<br>1. change rate of cardiovascular events [Time Frame: at least 18 months]<br>2. NT-proBNP [Time Frame: 12 months]<br>3. Kansas City Cardiomyopathy Questionnaire (KCCQ) [Time Frame: 1month, 6month,18 months]<br><br>(more details in the study.)	• Study Start: July 6, 2020<br><br>• Primary Completion: November 2022<br><br>• Study Completion: June 2023<br><br>• First Posted: October 1, 2020<br><br>• Results First Posted: No Results Posted<br><br>• Last Update Posted: October 8, 2020	• Shenzhen Second People's Hospital<br><br>• Funder type: Other	Shenzhen Second People's Hospital, Shenzhen, Guangdong, China	Evaluate the efficacy and safety of sacubitril/valsartan on cardiovascular events in patients with heart failure who are undergoing maintenance hemodialysis and peritoneal dialysis.

						5. Patients have a plasma N-terminal pro-BNP (NT-proBNP) level ≥600 pg per milliliter, or if they had been hospitalized for heart failure within the previous 12 months, an NT-proBNP ≥400 pg/mL.<br><br>(exclusion details present in the study.)						

3	• NCT number: NCT03771729 • Other Id's: 42411671-X	Observation on the Effect of Sacubitril/ Valsartan in Advanced Chronic Kidney Disease#CKD#Patients With Heart Failure	Unknown status	• CKD	Study Type: Interventional<br><br>Phase: Early Phase 1<br><br>•Allocation: N/A<br><br>•Intervention Model: Single Group Assignment<br><br>•Masking: None (Open Label)<br><br>•Primary Purpose: Treatment	•Enrollment: 30 participants.<br><br>•Age: 18 Years to 80 Years (Adult, Older Adult).<br><br>•Gender: All.<br><br>Inclusions Criteria:<br><br>•patients diagnosed with Chronic kidney disease (eGFR<60 ml/ min/1.73m²) and heart failure.<br>•Documented history of heart failure with associated signs or symptoms.<br>•New York Heart Association (NYHA) classes II-IV mean sitting systolic blood pressure (msSBP) ⩾140mmHg good compliance.<br><br>(exclusion details present in the study.)	Drug: LCZ 696	Primary Outcome:<br>•Change in estimated glomerular filtration rate(eGFR).<br>•Change in urinary microalbumin/creatinine ratio(uACR).<br>•Concentration of N terminal pro B type natriuretic peptide(NT-prpBNP).<br><br>Secondary Outcome:<br>•blood uric acid<br>•Rate of HbA1c<br>•Systolic and diastolic blood pressure<br>•Left ventricle eject fraction<br>•Concentration of postassium<br>•Concentration of serum troponin<br>•Concentration of alanine aminotransferase or aspartate aminotransferase<br>•Concentration of sodium	Study Start: December 30, 2018.<br><br>Primary Completion: May 28, 2019.<br><br>Study Completion: June 15, 2019.<br><br>First Posted: December 11, 2018.<br><br>Results First Posted: No Results Posted.<br><br>Last Update Posted: December 11, 2018.	• The Second Affiliated Hospital of Harbin Medical University.<br><br>•Funder type: Other	N/A.	This paper will mainly present the renal results from a clinical study aimed to observe the effect of sacubitril/valsartan in advanced chronic kedney disease patients with heart failure.

4	• NCT number: NCT05053893 • Other IDs: XHASB-001	Roxadustat Combined With Sacubitril Valsartan Sodium Tablets in the Treatment of Cardiorenal Anemia Syndrome	Recruiting	• Cardio-Renal Syndrome	• Study Type: Interventional<br><br>• Phase: Not Applicable<br><br>• Allocation: Randomized<br><br>• Intervention Model: Parallel Assignment<br><br>• Masking: None (Open Label)<br><br>• Primary Purpose: Treatment	• Enrollment: 40<br><br>• Ages: 18 Years to 70 Years<br><br>• Gender: All<br><br>• Inclusion:<br>- Diagnosed as regular dialysis patients with cardiorenal anemia syndrome<br><br>- Hemoglobin 60-110g / L (twice with an interval of at least 4 days);<br><br>- Volunteered to participate<br><br>(exclusion details present in the study.)	•Intervention: New treatment group(Roxadustat combined with Sacubitril Valsartan Sodium Tablets)<br><br>•Comparator: Traditional treatment group(EPO combined with ACEI or ARB)	• Primary outcome:<br>1. Changes of hemoglobin level before and after treatment [Time Frame: Day0-Day90]<br>2. Changes of ejection fraction before and after treatment [Time Frame: Day0-Day90]<br>3. Incidence of acute heart failure, acute myocardial infarction, severe hyperkalemia and severe anemia during treatment [Time Frame: Day0-Day90]<br><br>• Secondary outcome:<br>Not provided<br><br>(more details in the study.)	• Study start: September 22, 2021<br><br>• Primary Completion: July 1, 2022<br><br>• Study Completion: September 1, 2022<br><br>• First Posted: September 23, 2021<br><br>• Results First Posted: No Results Posted<br><br>• Last Update Posted: September 23, 2021	• The Affiliated Hospital of Qingdao University.<br>• Funder Type: Other	• Department of Nephrology, Affiliated Hospital of Qingdao University, Qingdao, Shangdong, China	
Infectious Disease												

1	• NCT number: NCT04153136<br><br>• Other Id's: MGH2019P002355	Effects of Sacubitril/Valsartan on Subclinical Heart Failure in HIV (The ENCHANTMENT HIV Study)	Recruiting	• HIV/AIDS<br>• Heart Failure With Preserved Ejection Fraction	Study Type: Interventional<br><br>Phase: Phase 2<br><br>•Allocation: Randomized<br><br>•Intervention Model: Parallel Assignment<br><br>•Masking: Quadruple (Participant, Care Provider, Investigator, Outcomes Assessor)<br><br>•Primary Purpose: Treatment	• Enrollment: 50<br><br>• Age: 40 Years to 70 Years (Adult, Older Adult)<br><br>• Sex: All<br><br>• Inclusion criteria:<br>1. Antiretroviral therapy use for >12 months<br><br>2. HIV Viral Load <200 copies/mL<br><br>3. Increased waist circumference based on NCEP criteria (male>102cm2 and female>88cm2) or increased waist to hip ratio based on WHO criteria (male>0.95 and female>0.80)<br><br>4. Left Ventricular Ejection Fraction>50%<br><br>5. Demonstration of one or more criteria for myocardial dysfunction	• Drug: Sacubitril-Valsartan 49-51Mg Oral Tablet<br><br>• Drug: Placebo oral tablet	• Primary outcome: [Time Frame: 6 months]<br>1. Myocardial Inflammation/Fibrosis.<br>2. Left Atrial Volume Index.<br><br>• Secondary outcome: [Time Frame: 6 months]<br>1. Indices of Myocardial Dysfunction.<br>2. Markers of Myocardial Inflammation and Fibrosis.<br>3. Cardiac Natriuretic Peptides.<br><br>(more details in the study.)	• Study Start: September 11, 2020<br><br>• Primary Completion: June 30, 2024<br><br>• Study Completion: December 31, 2024<br><br>• First Posted: November 6, 2019<br><br>• Results First Posted: No Results Posted<br><br>• Last Update Posted: October 15, 2021	• Massachusetts General Hospital<br><br>• Funder type: Other	Massachusetts General Hospital, Boston, Massachusetts, United States	The study aims to decrease HIV-related heart failure with a preserved ejection fraction with Sacubitril/valsartan.

						on cardiac transthoracic echocardiogram, relevant to the progression of heart failure with preserved ejection fraction:<br>- Left Atrial Volume Index > 28 mL/m2<br>- Global Longitudinal Strain <18%<br>- Left Ventricular Mass Index > 95g/m2 (female), 115 g/m2 (male)<br><br>(exclusion details present in the study.)						

2	• NCT number: NCT04023227<br><br>• Title Acronym: PARACHUTE-HF<br><br>• Other Ids: CLCZ696B3302	Efficacy and Safety of Sacubitril/ Valsartan Compared With Enalapril on Morbidity, Mortality, and NT-proBNP Change in Patients With CCC	Recruiting	•Chagas Disease<br><br>•Heart Failure	Study Type: Interventional<br><br>Phase: Phase 4<br><br>Study Design:<br>•Allocation: Randomized<br><br>•Intervention Model: Parallel Assignment<br><br>•Masking: Single (Outcomes Assessor)<br><br>•Primary Purpose: Treatment	Enrollment: 900 participants<br><br>Age: 18 Years and older (Adult, Older Adult)<br><br>Gender: All<br><br>Inclusion Criteria:<br><br>•Male or female ≥ 18 years of age<br>•Diagnosis of NYHA Class II-IV HFrEF established by<br>•Chagas' disease diagnosis confirmed by at least 2 different serological tests for anti-Trypanosoma cruzi ([ELISA], [IFI], [IHA]). (exclusion details present in the study.)	•Drug: Sacubitril/ valsartan<br><br>•Drug: Enalapril	Primary Outcome:<br>•Hierarchical composite endpoint composed of time to CV death, time to first HF hospitalization, relative change in NT-proBNP from baseline to Week 12<br><br>Secondary Outcome:<br>•Time to the first occurrence of a composite of CV events<br>•Time to all-cause mortality<br>•Time to sudden death or resuscitated sudden cardiac arrest<br>•Number of visits to an ER due to HF<br>•Number of days alive out of the hospital<br>•Number of ventricular fibrillation or sustained ventricular tachycardia<br>•Number of anti-tachycardia pacing or shock therapies	Study Start: December 10, 2019<br><br>Primary Completion: December 16, 2022<br><br>Study Completion: December 16, 2022<br><br>First Posted: July 17, 2019<br><br>Results First Posted: No Results Posted<br><br>Last Update Posted: April 1, 2021	•Novartis Pharmaceuticals<br><br>Funder Type:<br>•Industry	•Novartis Investigative Site, Rosario, Santa Fe, Argentina<br>•Novartis Investigative Site, Cordoba, Argentina<br>•Novartis Investigative Site, Cordoba, Argentina<br>•Novartis Investigative Site, Cordoba, Argentina<br>•Novartis Investigative Site, Santa Fe, Argentina<br>•Novartis Investigative Site, Salvador, BA, Brazil<br>•Novartis Investigative	The purpose of this study is to evaluate the effect of sacubitril/valsartan 200 mg BID compared with enalapril 10 mg BID, in addition to conventional heart failure (HF) treatment, in improving a hierarchical composite of cardiovascular (CV) events and causing a greater reduction in n terminal prohormone of brain natriuretic peptide (NT-proBNP, at Week 12 from Baseline) in participants with HF with reduced ejection

											Site, Campinas, SP, Brazil	fraction (HFrEF) caused by CCC.

3	• NCT number: NCT04853758<br><br>• Other IDs: 33360220500000068<br><br>• Title Acronym: ANSWER-HF	Angiotensin Receptor-Neprilysin Inhibition in Chagas Cardiomyopathy With Reduced Ejection Fraction	Recruiting	• Chagas Cardiomyopathy	• Study Type: Interventional<br><br>• Phase: Phase 3<br><br>• Allocation: Randomized<br><br>• Intervention Model: Parallel Assignment<br><br>• Masking: Triple (Participant, Investigator, Outcomes Assessor)<br><br>• Primary Purpose: Treatment	• Enrollment: 200<br><br>• Ages: 18 Years and older<br><br>• Gender: All<br><br>• Inclusion:<br>- Positive serology for Chagas;<br>- Age > 18 years old;<br>- New York Heart Association (NYHA) heart failure and functional class II, III or IV;<br>- Left ventricular ejection fraction <40% at least in the last 3 months;<br>- Patients using a beta-blocker with stable dose (last 4 weeks) and optimized;<br>-Patients using ACEI or ARB with a stable dose (last 4 weeks) and optimized.<br>(exclusion details present in the study.)	•Intervention: Sacubitril/Valsartan<br><br>•Comparator: Enalapril	• Primary outcome:<br>1. Change of left ventricular ejection fraction (LVEF) [Time Frame: 6 months]<br><br>• Secondary outcome:<br>1. Number of premature ventricular beats [Time Frame: 6 months]<br>2. Percentage of premature ventricular beats. [Time Frame: 6 months]<br>3. Ventricular arrhythmias density [Time Frame: 6 months]<br>4. Sustained ventricular tachycardia rates [Time Frame: 6 months]<br>5. New York Heart Association functional class [Time Frame: 6 months]<br>6. Ventricular remodeling - left ventricular systolic diameter [Time Frame: 6 months]<br><br>(more details in the study.)	• Study start: May 6, 2021<br><br>• Primary Completion: March 1, 2023<br><br>• Study Completion: March 1, 2023<br><br>• First Posted: April 21, 2021<br><br>• Results First Posted: No Results Posted<br><br>• Last Update Posted: May 7, 2021	• University of Sao Paulo General Hospital<br><br>• nCor Heart Institute.<br>• Funder Type: Other	• Heart Institute (Incor) University of Sao Paulo, São Paulo, Brazil	
Transplant												

1	• NCT number: NCT04092309<br><br>• Other Ids: ACE_SAVA_3Decho_BMT	Effect of Angiotensin Converting Enzyme and Sacubitril Valsartan in Patients After Bone Marrow Transplantation	Recruiting	•Hematopoietic Stem Cell Transplantation<br><br>•Cardiotoxicity	Study Type: Interventional<br><br>Phase: Not Applicable<br><br>Study Design:<br>•Allocation: Randomized<br><br>•Intervention Model: Parallel Assignment<br><br>•Masking: None (Open Label)<br><br>•Primary Purpose: Prevention	Enrollment: 90 Participant<br><br>Age: 18 Years and older (Adult, Older Adult)<br><br>Gender: All<br><br>Inclusion Criteria:<br>•Adolescents male and female that have just been treated with bone marrow transplantation due to haematological malignancies. (exclusion details present in the study.)	•Drug: ACE inhibitor, Sacubitril - Valsartan	Primary Outcome Measures :<br>•Effect of treatment in Left Ventricular Function<br>•Effect of treatment in left ventricular function<br>•Effect of treatment in arterial stiffness<br>•Effect of treatment in glycocalyx thickness	Study Start: September 20, 2019<br><br>Primary Completion: September 1, 2020<br><br>Study Completion: September 1, 2021<br><br>First Posted: September 17, 2019<br><br>Results First Posted: No Results Posted<br><br>Last Update Posted: April 3, 2020	•University of Athens<br><br>Funder Type:<br>•Other	•"Attikon" University General Hospital, Athens, Attiki, Greece	The purpose of the present study is to investigate the effect of ACE inhibitors and the sacubitril-valsartan complex in bone marrow transplant patients by assessing cardiovascular and endothelial parameters in order to search for a potent protective role.
Cognitive													

1	• NCT number: NCT05117736<br><br>•Other Study ID Numbers: CLCZ696B2320<br><br>•Title Acronym: PERSPECTIVE	Efficacy and Safety of LCZ696 Compared to Valsartan on Cognitive Function in Patients With Chronic Heart Failure and Preserved Ejection Fraction (PERSPECTIVE)	Active, not recruiting	• Chronic Heart Failure (CHF)	•Study Type: Interventional<br><br>•Phase: Phase 3<br><br>•Study Design:<br><br>•Allocation: Randomized<br><br>•Intervention Model: Parallel Assignment<br><br>•Masking: Double (Participant, Investigator)<br><br>•Primary Purpose: Treatment	•Enrollment : 592<br><br>•Age:60 Years and older<br><br>•Gender: All<br><br>•Inclusion Criteria:<br>1. Chronic heart failure with current symptoms NYHA class II-IV<br><br>2. Left ventricular ejection fraction > 40%<br><br>3. NT-proBNP >= 125 pg/mL at screening visit<br><br>4. Patient with evidence of adequate functioning to complete study assessments<br><br>(exclusion details present in the study.)	•Intervention-Experimental: LCZ696 Patients will receive LCZ696 at 100 mg twice daily<br><br>•Active Comparator: Valsartan Patients will receive valsartan at 40mg and/or 80mg twice daily	•Primary Outcome: [Time Frame: Baseline, week 156]<br>1. Change from baseline in the CogState Global Cognitive Composite Score (GCCS)<br><br>• Secondary outcome: [Time Frame: Baseline, week 156]<br>1. Change from baseline in cortical composite standardized uptake value ratio (SUVr)<br><br>2. Change from baseline in individual cognitive domains (memory, executive function, and attention)<br><br>3. Change from baseline in the summary score of the instrumental activities of daily living (IADL)	•Study Start: November 23, 2016<br><br>•Primary Completion: March 28, 2022<br><br>•Study Completion: March 28, 2022<br><br>•First Posted: August 30, 2016<br><br>•Results First Posted: No Results Posted<br><br>• Last Update Posted: November 17, 2021	• Novartis Pharmaceuticals<br><br>•Funder type: Industry	Novartis Investigative Site, Andalusia, Alabama, United States<br>•Novartis Investigative Site, Glendale, Arizona, United States<br>•Novartis Investigative Site, Mesa, Arizona, United States<br>•Novartis Investigative Site, Phoenix, Arizona, United States<br>•Novartis Investigative Site, Sun City West, Arizona, United States<br>•Novartis Investigative Site, Tucson, Arizona, United States	The purpose of this study is to evaluate the effect of LCZ696 compared to valsartan on cognitive function in patients with chronic heart failure and preserved ejection fraction. Cognitive function will be assessed using a comprehensive battery of tests with an evaluation of longitudinal change of cognitive domains including memory, executive function, and attention.

											•Novartis Investigative Site, Beverly Hills, California, United States •Novartis Investigative Site, Fresno, California, United States •Novartis Investigative Site, Loma Linda, California, United States •Novartis Investigative Site, Long Beach, California, United States •and 126 more	
Pediatric												

1	• NCT number: NCT03785405 • Other Id's: CLCZ696B2319E1	CLCZ696B2319E1 OL Extension Study to Evaluate Long-term Safety of Sacubitril/ Valsartan in Pediatric Patients With HF	Recruiting	• Heart Failure	• Study Type: Interventional<br><br>• Phase: Phase 3<br><br>• Allocation: N/A<br><br>• Intervention Model: Parallel Assignment<br><br>• Masking: None (Open Label)<br><br>• Primary Purpose: Treatment	• Enrollment: 240<br><br>• Age:1 Year to 18 Years (Child, Adult)<br><br>• Sex: All<br><br>• Inclusion criteria:<br>1. Signed informed consent.<br><br>2. No safety issues on study drug at PANORAMA-HF Part 2 EOS visit.<br><br>(exclusion details present in the study.)	• Drug: sacubitril / valsartan	• Primary outcome: [Time Frame: to end of study, up to 3 years]<br>1. Number of participants with Adverse Events (AEs) as a measure of safety and tolerability.<br>2. Number of participants with Serious Adverse Events (SAEs) as a measure of safety and tolerability.<br><br>(more details in the study.)	• Study Start: May 2, 2019<br><br>• Primary Completion: April 21, 2022<br><br>• Study Completion: December 31, 2022<br><br>• First Posted: December 24, 2018<br><br>• Results First Posted: No Results Posted<br><br>• Last Update Posted: December 6, 2021	• Novartis Pharmaceuticals<br>• Novartis<br><br>• Funder type: Other	•Novartis Investigative Site, Loma Linda, California, United States<br><br>•Novartis Investigative Site, Los Angeles, California, United States<br><br>•Novartis Investigative Site, Palo Alto, California, United States<br><br>•Novartis Investigative Site, Hollywood, Florida, United States<br><br>•Novartis Investigative Site, Saint Petersburg, Florida, United	Investigate the long-term safety and tolerability of sacubitril/valsartan in eligible CLCZ696B2319 (PANORAMA-HF) patients.

											States<br><br>•Novartis Investigative Site, Atlanta, Georgia, United States<br><br>•Novartis Investigative Site, Indianapolis, Indiana, United States<br><br>•Novartis Investigative Site, Boston, Massachusetts, United States<br><br>•Novartis Investigative Site, Ann Arbor, Michigan, United States<br><br>•Novartis Investigative Site, Minneapolis, Minnesota, United States	

											•and 66 more	

2	• NCT number:NCT02678312<br><br>• Other Study ID Numbers: CLCZ696B2319<br><br>2015-004207-22 (EudraCT Number)	Study to Evaluate Safety, Tolerability, Pharmacokinetics and Pharmacodynamics of LCZ696 Followed by a 52-week Study of LCZ696 Compared With Enalapril in Pediatric Patients With Heart Failure	Active, not recruiting	•Heart Failure (Pediatric)	•Study Type: Interventional<br><br>•Phase: Phase2 Phase 3<br><br>•Allocation: Randomized<br><br>•Intervention Model: Parallel Assignment<br><br>•Masking: Double (Participant, Investigator)<br><br>•Primary Purpose: Treatment	•Enrollment: 393<br><br>•Ages: 1 Month to 17 Years (Child)<br><br>•Gender: All<br><br>•Inclusion Criteria: 1. Chronic heart failure resulting from left ventricular systolic dysfunction, and receiving chronic HF therapy (if not newly diagnosed)<br><br>2. NYHA classification II-IV (older children: 6 to <18 years old) or Ross CHF classification II-IV (younger children: < 6 years old)<br><br>3. Systemic left ventricular ejection fraction ≤ 40% or fractional shortening ≤20%<br><br>4. For Part 1 study: Patients must be treated with an ACEI or ARB prior	•Intervention<br>•Experimental: Part 1: LCZ696 LCZ696 open label either 1) 0.8 mg/kg or 2) 3.1 mg/kg or both. After LCZ696 PK assessment, patients will be maintained on open-label Enalapril or standard of care for heart failure treatment, if patient consents to participate in Part 2.<br><br>•Active Comparator: Part 2: Enalapril The target dose for enalapril is 0.2 mg/kg bid (0.4 mg/kg total daily dose) with a	• Primary outcome:<br>Part 1 of the study:<br>1. Pharmacokinetics of LCZ696 analytes (sacubitril, LBQ657, and valsartan): Maximum drug concentration in plasma (Cmax)<br>2. Pharmacokinetics of LCZ696 analytes (sacubitril, LBQ657, and valsartan): Time to maximum plasma concentration (Tmax)<br>3. Pharmacokinetics of LCZ696 analytes (sacubitril, LBQ657, and valsartan): area under the plasma concentration- time curve from time zero to infinity (AUCinf) and area under the plasma concentration-time curve from time zero to last (AUClast)<br>4. Pharmacokinetics of LCZ696 analytes (sacubitril, LBQ657, and valsartan): Clearance from plasma (CL/F)<br>5. Pharmacokinetics of LCZ696 analytes (sacubitril, LBQ657, and valsartan): Time required to drug concentration to decrease by half (T 1/2)<br>6. Pharmacodynamics of LCZ696 analytes (sacubitril, LBQ657, and valsartan): Plasma N-terminal pro-brain natriuretic peptide (NTproBNP)<br>7. Pharmacodynamics of LCZ696 analytes (sacubitril, LBQ657, and valsartan): Plasma cyclic guanosine monophosphate (cGMP)<br>8. Pharmacodynamics of LCZ696 analytes (sacubitril, LBQ657, and valsartan): Urine cGMP<br>9. Pharmacodynamics of LCZ696 analytes (sacubitril, LBQ657, and valsartan): Plasma B-type natriuretic peptide (BNP)<br><br>Part 2 of the study:[Time Frame: Up to 52 weeks]<br>10. Percentage of patients falling into each category based on global ranking<br><br>• Secondary outcome: [Time Frame: 52 weeks]<br>Part 2 of the study:<br>1. Time to first occurrence of Category 1 or Category 2 event<br>2. Change from baseline in NYHA/Ross functional class<br>3. Change from baseline in Patient Global impression of severity score (PGIS) scale<br>4. Population PK of LCZ696 analytes (sacubitril, LBQ657, and valsartan): Clearance from plasma in steady state (CL,ss)<br>5. Population PK of LCZ696 analytes (sacubitril, LBQ657, and valsartan): Volume of distribution in steady state<br>6. Population PK of LCZ696 analytes (sacubitril, LBQ657, and valsartan): Absorption rate constant in steady state (Ka,ss)<br>7. Population PK of LCZ696 analytes (sacubitril, LBQ657, and valsartan): Time required to drug concentration to decrease by half in steady state (T 1/2,ss)<br>8. Population PK of LCZ696 analytes (sacubitril, LBQ657, and valsartan): Maximum drug	• Study Start: November 3, 2016<br><br>• Primary Completion: December 31, 2021<br><br>•Study Completion: December 31, 2021<br><br>•First Posted: February 9, 2016<br><br>• Results First Posted: No Results Posted<br><br>• Last Update Posted: October 28, 2021	• Novartis Pharmaceuticals<br><br>•Funder type: Industry	Novartis Investigative Site, Andalusia, Alabama, United States<br>•Novartis Investigative Site, Glendale, Arizona, United States<br>•Novartis Investigative Site, Mesa, Arizona, United States<br>•Novartis Investigative Site, Phoenix, Arizona, United States<br>•Novartis Investigative Site, Sun City West, Arizona, United States<br>•Novartis Investigative Site, Tucson, Arizona, United States	This study consist of two parts (Part 1 and Part 2). The purpose of Part 1 is to evaluate the way the body absorbs, distributes and removes the drug LCZ696. This will help determine the proper dose of LCZ696 for Part 2 of the study.<br><br>The purpose for Part 2 is to compare the effectiveness and safety of LCZ696 with enalapril in pediatric heart failure patients over 52 weeks of treatment.

						to screening. Patients in Group 1 and 2 must be currently treated with the dose equivalent of at least enalapril 0.2 mg/kg prior to the LCZ696 3.1 mg/kg administration. Group 3 patients will participate in LCZ696 0.8 mg/kg and not LCZ696 3.1 mg/kg.<br><br>5. Biventricular physiology with systemic left ventricle<br><br>(exclusion details present in the study.)	maximum dose of 10 mg bid (20 mg total daily dose).<br><br>•Experimental: Part 2:LCZ696 LCZ696 3.125 mg granules and adult formulation (50, 100, 200 mg) can be given based on patient weight.	concentration in plasma at steady state (Cmax,ss)<br>9. Population PK of LCZ696 analytes (sacubitril, LBQ657, and valsartan): Lowest plasma concentration observed during a dosing interval at steady state (Cmin,ss)<br>10. Population PK of LCZ696 analytes (sacubitril, LBQ657, and valsartan): area under the plasma concentration-time curve from time zero to the end of the dosing interval tau at steady state (AUCtau,ss)			•Novartis Investigative Site, Beverly Hills, California, United States<br>•Novartis Investigative Site, Fresno, California, United States<br>•Novartis Investigative Site, Loma Linda, California, United States<br>•Novartis Investigative Site, Long Beach, California, United States<br>•and 126 more	
Pharmacodynamics and Pharmacokinetics												

1	• NCT number:NCT04649229<br><br>•Other Ids: 2000028712	Mechanisms Underlying Hypotensive Response to ARB/NEP Inhibition - Aim 3	Recruiting	•Heart Failure	•Study Type: Interventional<br><br>•Phase: Phase 4<br><br>•Allocation: Randomized<br><br>•Intervention Model: Crossover Assignment<br><br>•Masking: Quadruple (Participant, Care Provider, Investigator, Outcomes Assessor)<br><br>•Primary Purpose: Basic Science	•Enrollment: 80<br><br>•Age: 18 Years and older<br><br>• Gender: All<br><br>•Inclusion Criteria:<br>1.Black and white men and women<br>2.Stable patients with a reduced ejection fraction (EF)<br>A.EF ≤55%, and<br>B.history of symptoms of New York Heart Association (NYHA) class I, II, or III heart failure (HF)<br>C.stable clinical symptoms including no hospitalizations for the last three months, or one month if hospitalized only once for initial diagnosis of HF<br>D. who are not already taking LCZ696<br>3. treatment with a stable dose of an angiotensin-converting	•Drug: LCZ 696<br>•Drug: Placebo<br>•Drug: Para-aminohippurate<br>•Drug: Iohexol<br>•Drug: Aprepitant	• Primary outcome:<br>1. Mean arterial pressure (MAP) [Time Frame: Over six hours on each of the four study days]<br>2. Urine sodium excretion [Time Frame: Total urine output from drug administration to six hours following drug administration]<br><br>• Secondary outcome: [Time Frame: Over six hours on each of four study days]<br>1. Heart rate<br>2.Urine volume<br>3.Renal plasma flow<br>4.Glomerular filtration rate<br>5. Urine albumin-to-creatinine ratio	•Study Start: May 27, 2021<br><br>•Primary Completion: July 2023<br><br>•Study Completion: January 2024<br><br>•First Posted: December 2, 2020<br><br>•Results First Posted: No Results Posted<br><br>•Last Update Posted: December 1, 2021<br>•Yale University<br><br>•Funder type: Other	•Yale University<br><br>•Funder type: Other	•Yale New Haven Hospital, New Haven, Connecticut, United States	This is a double-blind, randomized, two x two crossover (aprepitant vs placebo) during both initiation of Entresto, LCZ696, (50 mg dose) and at steady-state of Entresto (200 mg bid dose or the highest tolerated dose).

						enzyme inhibitor (ACEi) or angiotensin receptor blocker (ARB) and with a beta blocker (unless contraindicated or not tolerated) for at least four weeks<br>4. for patients with NYHA Class II or III HF, treatment with a stable dose of an mineralocorticoid receptor (MR) antagonist for at least four weeks, unless not possible due to renal function or adverse reaction<br>5. For female subjects, the following conditions must be met:<br><br>A.postmenopausal status for at least one year<br>B. status post-surgical sterilization<br>C. if childbearing potential,							

						utilization of barrier methods of birth control or an oral contraceptive and willingness to undergo urine β-HCG testing on every study day<br>6. Age 18 years of age or older (exclusion details present in the study.)						

2	• NCT number: NCT04113109<br><br>• Other Ids: #191228	Mechanisms Underlying Hypotensive Response to ARB/NEP Inhibition - Aim 2	Recruiting	• Heart Failure	•Study Type: Interventional<br><br>•Phase: Not Applicable<br><br>•Allocation: Randomized<br><br>•Intervention Model: Parallel Assignment<br><br>•Masking: None (Open Label)<br><br>•Primary Purpose: Treatment	•Enrollment: 80<br><br>•Age: 18 Years and older<br><br>• Gender: All<br><br>•Inclusion Criteria:<br>1.Black and white men and women<br>2.Stable patients with a reduced ejection fraction (EF)<br>A.EF ≤55%, and<br>B.history of symptoms of New York Heart Association (NYHA) class I, II, or III heart failure (HF)<br>C.stable clinical symptoms including no hospitalizations for the last three months, or one month if hospitalized only once for initial diagnosis of HF<br>D. who are not already taking LCZ696<br>3. treatment with a stable dose of an angiotensin-converting	•Drug: LCZ 696<br>•Drug: Placebo<br>•Drug: Para-aminohippurate<br>•Drug: Iohexol<br>•Drug: Aprepitant	• Primary outcome:<br>1. Mean arterial pressure (MAP) [Time Frame: Over six hours on each of the four study days]<br>2. Urine sodium excretion [Time Frame: Total urine output from drug administration to six hours following drug administration]<br><br>• Secondary outcome: [Time Frame: Over six hours on each of four study days]<br>1. Heart rate<br>2.Urine volume<br>3.Renal plasma flow<br>4.Glomerular filtration rate<br>5. Urine albumin-to-creatinine ratio	•Study Start: November 1, 2019<br><br>•Primary Completion: April 2023<br><br>•Study Completion: October 2023<br><br>•First Posted: October 2, 2019<br><br>•Results First Posted: No Results Posted<br><br>•Last Update Posted: April 27, 2021	• Vanderbilt University Medical Center<br><br>•Funder type: Other	Vanderbilt University Medical Center, Nashville, Tennessee, United States	The main objectives of this mechanistic randomized, double-blind, crossover-design study are:<br><br>The primary objective is to test the hypothesis that endogenous bradykinin contributes to effects of ARB/NEP inhibition on blood pressure, natriuresis, and diuresis at initiation. The secondary objective is to test the hypothesis is endogenous bradykinin contributes to effects of ARB/NEP inhibition

						enzyme inhibitor (ACEi) or angiotensin receptor blocker (ARB) and with a beta blocker (unless contraindicated or not tolerated) for at least four weeks<br>4. for patients with NYHA Class II or III HF, treatment with a stable dose of an mineralocorticoid receptor (MR) antagonist for at least four weeks, unless not possible due to renal function or adverse reaction<br>5. For female subjects, the following conditions must be met:<br><br>A.postmenopausal status for at least one year<br>B. status post-surgical sterilization<br>C. if childbearing potential,						on blood pressure, natriuresis, and diuresis after up-titration.

						utilization of barrier methods of birth control or an oral contraceptive and willingness to undergo urine β-HCG testing on every study day<br><br>(exclusion details present in the study.)						

3	•NCT number: NCT03553303<br><br>•Other Study ID Numbers: 3403003	Pharmacodynamic Effects of Sacubitril/ Valsartan on Natriuretic Peptides, Angiotensin and Neprilysin	Recruiting	• Heart Failure, Systolic	• Study Type: Interventional<br><br>• Phase: Phase 4<br><br>• Allocation: N/A<br><br>• Intervention Model: Single Group Assignment<br><br>• Masking: None (Open Label)<br><br>• Primary Purpose: Treatment	• Enrollment: 40<br><br>• Ages: Child, Adult, Older Adult<br><br>• Gender: All<br><br>• Inclusion: 1.Patients must give written informed consent before any study assessment is performed. 2.Ambulatory ≥ 18 years of age, male or female, treated at Ringerike Hospital. 3.Patients with symptomatic chronic heart failure and reduced ejection fraction (≤ 40%). 4.Patients on optimized medical treatment for heart failure.<br><br>•Exclusion Criteria: 1.Patients not able to comply in the study. 2.Patients having contraindic	•Intervention Increasing doses of Sacubitril/Valsartan	• Primary outcome:<br>1.Neurohormonal plasma concentration [Time Frame: 8 weeks]	•Study Start: October 16, 2018<br><br>•Primary Completion: May, 2022<br><br>•Study Completion: May, 2022<br><br>•First Posted: June 12, 2018<br><br>•Results First Posted: No Results Posted<br><br>•Last Update Posted: August 29, 2019	Oslo University Hospital<br><br>•Funder Type: Other	•Ringerike Hospital Vestre Viken Hospital Trust, Hønefoss, Buskerud, Norway	The study measure multiple neurohormones in patients with heart failure being treated with Sacubitril/Valsartan in increasing doses over an 8 week period.

						ation for treatment with Entresto;<br>A.Hypersensitivity to the active substances or to any of the excipients listed in section<br>B.Hyperkalemia: > 5.4 mmol/L<br>C.Known history of angioedema related to previous ACE inhibitor or ARB therapy.<br>D.Hereditary or idiopathic angioedema.<br>E.Concomitant use with Aliskiren-containing medicinal products in patients with diabetes mellitus or in patients with renal impairment (eGFR <60 ml/min/1.73 m2)<br>F.End-stage renal disease (<15 mL/min per 1.73m2 or treatment by dialysis).<br>G.Severe hepatic impairment, biliary						

						cirrhosis and cholestasis (Child-Pugh C classification). H.Pregnancy Breast-feeding						

4	• NCT number: NCT03738878<br><br>•Other Ids: IRB#170762	Mechanism(s) Underlying Hypotensive Response to ARB/NEP Inhibition - Aim 1	Active, not recruiting	• Hypertension	•Study Type: Interventional<br><br>•Phase: Phase 4<br><br>•Allocation: Randomized<br><br>•Intervention Model: Crossover Assignment<br><br>•Masking: Quadruple (Participant, Care Provider, Investigator, Outcomes Assessor)<br><br>•Primary Purpose: Basic Science	•Enrollment: 32<br><br>•Age: 18 Years to 60<br><br>• Gender: All<br><br>Inclusion Criteria:<br><br>1.Patients with essential hypertension defined as having<br><br>A. untreated, seated systolic blood pressure (SBP) of 130 mmHg or greater on three separate occasions, or<br>B. untreated, seated diastolic BP (DBP) of 80 or greater on three separate occasions, or<br>C. taken anti-hypertensive agent(s) for a minimum of six months.<br><br>2.For female subjects, the following conditions must be met:	•Drug: Valsartan<br>•Drug: LCZ696<br>•Drug: Bradykinin<br>•Drug: Substance P<br>•Drug: BNP<br>•Drug: Sitagliptin	• Primary outcome: [Time Frame: After four-week treatment with each crossover drug]<br>1.forearm blood flow<br>2. tissue-type plasminogen activator release<br><br>• Secondary outcome: [Time Frame: After four-week treatment with each crossover drug]<br>1.norepinephrine release	•Study Start:<br><br>November 15, 2018<br><br>•Primary Completion:<br>July 1, 2024<br><br>•Study Completion:<br>December 31, 2024<br><br>•First Posted: November 13, 2018<br><br>Results First Posted: No Results Posted<br><br>Last Update Posted: April 27, 2021	• Vanderbilt University Medical Center<br><br>•Funder type: Other	Vanderbilt University Medical Center, Nashville, Tennessee, United States	The purpose of this study is to test the hypothesis that combined angiotensin receptor blockade (ARB)/neprilysin (NEP) inhibition potentiates the effects of exogenous bradykinin, substance P, and brain natriuretic peptide (BNP) on forearm blood flow or endothelial tissue-type plasminogen activator (t-PA) release compared to ARB alone. A secondary goal is to determine if there is an interactive effect of ARB/NEP inhibition and

						A. postmenopausal status for at least one year, or<br>B. status post-surgical sterilization, or<br>C. if of childbearing potential, utilization of adequate birth control and willingness to undergo urine beta-human chorionic gonadotropin (hCG) testing prior to drug treatment and on every study day.						dipeptidyl peptidase 4 (DPP4) inhibition on responses to these peptides.
